# Enriching the Arsenal of Pharmacological Tools against MICAL2

**DOI:** 10.3390/molecules26247519

**Published:** 2021-12-11

**Authors:** Ivana Barravecchia, Elisabetta Barresi, Camilla Russo, Francesca Scebba, Chiara De Cesari, Valerio Mignucci, Davide De Luca, Silvia Salerno, Valeria La Pietra, Mariateresa Giustiniano, Sveva Pelliccia, Diego Brancaccio, Greta Donati, Federico Da Settimo, Sabrina Taliani, Debora Angeloni, Luciana Marinelli

**Affiliations:** 1 Institute of Life Sciences, Scuola Superiore Sant’Anna, Via G. Moruzzi 1, 56124 Pisa, Italy; ivana.barravecchia@santannapisa.it (I.B.); francesca.scebba@santannapisa.it (F.S.); chiara.decesari@biologia.unipi.it (C.D.C.); valerio.mignucci@santannapisa.it (V.M.); davide.deluca@santannapisa.it (D.D.L.); 2 Department of Pharmacy, University of Pisa, Via Bonanno 6, 56126 Pisa, Italy; elisabetta.barresi@unipi.it (E.B.); salerno@farm.unipi.it (S.S.); federico.dasettimo@farm.unipi.it (F.D.S.); 3 Dipartimento di Farmacia, Università degli Studi di Napoli Federico II, Via D. Montesano 49, 80131 Naples, Italy; camilla.russo2@unina.it (C.R.); valeria.lapietra@unina.it (V.L.P.); mariateresa.giustiniano@unina.it (M.G.); sveva.pelliccia@unina.it (S.P.); diego.brancaccio@unina.it (D.B.); greta.donati@unina.it (G.D.)

**Keywords:** MICAL2, wound healing assay, HMEC-1 endothelial cells, 786-O kidney cancer cells, CCG-1423, metastasis, neoangiogenesis, multicomponent reactions (MCRs), Passerini 3-CR reaction, Passerini-like 3-CR

## Abstract

Molecule interacting with CasL 2 (MICAL2), a cytoskeleton dynamics regulator, are strongly expressed in several human cancer types, especially at the invasive front, in metastasizing cancer cells and in the neo-angiogenic vasculature. Although a plethora of data exist and stress a growing relevance of MICAL2 to human cancer, it is worth noting that only one small-molecule inhibitor, named CCG-1423 (1), is known to date. Herein, with the aim to develop novel MICAL2 inhibitors, starting from CCG-1423 (1), a small library of new compounds was synthetized and biologically evaluated on human dermal microvascular endothelial cells (HMEC-1) and on renal cell adenocarcinoma (786-O) cells. Among the novel compounds, **10** and **7** gave interesting results in terms of reduction in cell proliferation and/or motility, whereas no effects were observed in MICAL2-knocked down cells. Aside from the interesting biological activities, this work provides the first structure–activity relationships (SARs) of CCG-1423 (1), thus providing precious information for the discovery of new MICAL2 inhibitors.

## 1. Introduction

**Figure 1:**
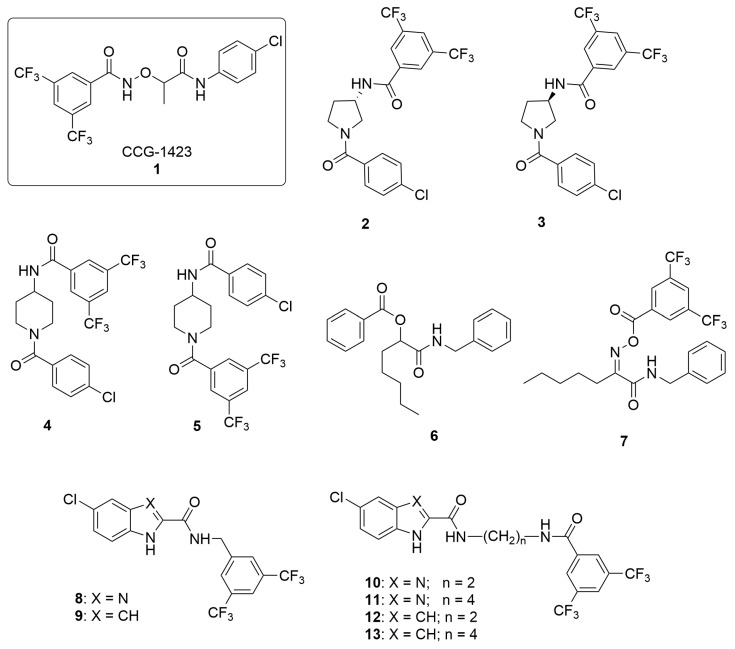
Chemical Structure of CCG-1423 and molecules herein presented.

MICAL2 (Molecule Interacting with CasL 2) is a multidomain nucleocytoplasmic protein belonging to the MICALs family. In *Homo sapiens*, this family consists of three members (MICAL1, MICAL2 and MICAL3) and two MICAL-like homologs (MICAL-L1 and MICAL-L2) [1,2,3]. MICAL proteins are redox enzymes that exert a dynamic control over polymerization of actin, one of the most abundant proteins in eukaryotic cells that plays an essential role in basic cell functions ranging from cell shape, adhesion, and motility to proliferation, differentiation, and survival. As such, MICALs are involved in key physiological functions such as cytoskeleton remodeling, vesicle trafficking, axon guidance, autophagy and phagocytosis, and angiogenesis. The structural organization of MICALs clearly reflects some of these functions [1]. In fact, they are made up of a N-terminal flavoprotein mono-oxygenase (MO) domain, required for semaphorin-plexin-mediated axon guidance [4,5], connected to other modules that network with cytoskeletal and signaling partners: a calponin homology (CH) domain, a LIM domain, and a SH3 domain-binding motif that mediates interaction with CasL, a multidomain docking protein localized at focal adhesions and stress fibers [4,5]. MICAL proteins are expressed in specific neuronal and nonneuronal tissues, during development as well as in adulthood. In neurons, MICAL2 induces redox-dependent depolymerization of nuclear actin that in turn decreases nuclear G-actin and increases MRTF-A concentration in the nucleus [6]. Up to now, although MICAL2 role in many human diseases is far from being clear, it is known to exert significant biological impacts on multiple cancer types. Some of us have previously demonstrated that MICAL2 is a new prometastatic gene, expressed in a variety of solid metastatic human tumors [7]. In fact, MICAL2 is involved in bladder cancer pathogenesis [8], in chemoresistance and increased mortality in endometrial cancer patients [9], and it is over-expressed in aggressive human gastric and kidney cancers [10]. Moreover, recent evidence unveiled the involvement of MICAL2 in cancer cell migration [11]. In particular, in breast cancer, cell migration is potentiated through maintaining epidermal growth factor receptor (EGFR) stability and activating EGFR/P38 signaling, whereas in gastric cancer cells the MICAL2/MRTF-A complex promotes migration through the CDC42 pathway [11]. These data make MICAL2 a valuable target against cancer cell migration, motility, and metastasis. Nowadays, drugs targeting these mechanisms are called migrastatics [12] and are particularly sought after by pharmaceutical industries. Further, we also reported that MICAL2 is expressed in neo-angiogenic endothelial cells (ECs) in human solid tumors (gastric, kidney and breast carcinoma, glioblastoma, and cardiac myxoma), and in animal models of ischemia/inflammation neo-angiogenesis, but not in the normal capillary bed [13]. Specifically, through immunohistochemistry, the expression of the MICAL2 protein was highlighted in ECs of neo-angiogenic capillaries branching off within the tumor mass of gastric, kidney, and breast carcinoma, glioblastoma, and cardiac myxoma [13]. Interestingly, ECs of newly formed capillaries were characterized by strong MICAL2 expression; therefore, they were distinct from ECs of preformed arterioles. The analysis of the MICAL2 transcriptome [13] in MICAL2 KD cells, revealed that MICAL2 is involved in angiogenesis and vascular development pathways. Most interestingly, its pharmacological inhibition (CCG-1423) or gene knock-down (KD) reduced EC viability and functional properties, hampering their ability to respond to VEGF stimulation. The anti-angiogenic approach to treat cancer, initially proposed by Judah Folkman [14], aims at controlling tumor growth through preventing the maturation of a functional vessel network, compromising the existing tumor-associated vasculature and inhibiting new vessel formation. Although current anti-angiogenic drugs allow in some cases impressive clinical achievements in terms of tumor shrinkage and increased survival, primary resistance and temporary efficacy remain a problem, calling for new approaches. Thus, all the aforementioned observations suggest MICAL2 not only as a target against cell-migration but also for anti-angiogenic therapy. Despite the growing number of data stressing the relevance of MICAL2 to human cancer, it is worth noting that CCG-1423 (**1**, Figure 1), namely a small molecule that inhibits the SRF/MRTF-A pathway, is the only MICAL2 inhibitor known to date. At this regard, the treatment of human dermal microvascular ECs (HMEC-1) with 10 μM CCG-1423 (**1**, Figure 1), led to a significant reduction of cell proliferation (70%) and of the covered area (60%) in an 6 h scratch or wound healing assay (WHA). Furthermore, **1** caused a complete containment of the cell motility response to TNF-alpha, as indicated by the absence of statistically significant difference between cells treated with CCG-1423 (**1**, Figure 1) before and after exposure to TNF-alpha [13].

WHA is one of the most commonly used bioassays for evaluating the therapeutic impact on cell migration, mainly due to its simplicity in experimental setup and data processing. By scratching a cell monolayer to create a wound, one can consistently perform WHA across a large number of treatments [15].

Herein, with the aim to enlarge the arsenal of pharmacological tools to inhibit MICAL2, starting from CCG-1423 molecular structure (**1**, Figure 1), a small library of diverse analogues was synthetized and biologically evaluated on different MICAL2-expressing ECs and on cancer cell lines in which MICAL2 gene was knocked down [7]. Based on the rationale above [12], we started testing our newly synthetized compounds with a simple viability assay based on Trypan blue exclusion assay [16], and 2D motility test (WHA) for possible effects on cell motility, to evaluate a perspective therapeutic impact on cancer cell invasion and neo-angiogenesis in vitro.

## 2. Results and Discussion

### 2.1. Design and Synthesis

To the best of our knowledge, any structure–activity relationships (SARs) of CCG-1423 (**1**, Figure 1) as MICAL2 inhibitor are available to date. In the case of MRTF-A protein binding, little data have been published and a preference for the S-isomer has been demonstrated.

Herein, based on the structure of CCG-1423 (**1**, Figure 1), we designed different rigidified analogues (**2**–**13**) that basically maintain the 3,5-bis(trifluoromethyl)phenyl and 4-chlorophenyl of the lead or feature aromatic ring able to mimic the two aryl moieties of **1**, and present variability in the linker. Specifically, derivatives **2-5** feature a *N*-carboxypyrrolidine (**2** and **3**, Figure 1), or *N*-carboxypiperidine (**4** and **5**, Figure 1) as central nuclei in which a portion of the original **1** bridge (-OCH(CH_3_)CONH) is included and link the 3,5-bis(trifluoromethyl)benzamide to a 4-chlorophenyl: in compounds **2** and **3,** the different stereochemistry at 3-position of the pyrrolidine was also investigated; in derivatives **4** and **5,** the relative position of the two aryl portions was exploited. In compounds **6** and **7** (Figure 1), the two aryl groups are connected by open-chain linkers, that are characterized by ester, amido, and oxyimino functions and support a *n*-pentane group in place of the methyl group of **1**, in order to explore the chemical space around the central position. Finally, in compounds **8**–**13** (Figure 1), the *p*-chlorophenylaminocarbonyl portion of **1** is included in the indole or benzimidazole nucleus, whereas the 3,5-bis(trifluoromethyl)phenyl is retained. In order to confer some adaptability to the whole molecule and in turn allow a better interaction with the target protein, a linker of variable length has been inserted, featuring one (**8** and **9**, Figure 1) or two (**10**–**13**, Figure 1) amido functions connecting the two pharmacophoric aromatic nuclei.

**Scheme 1:**
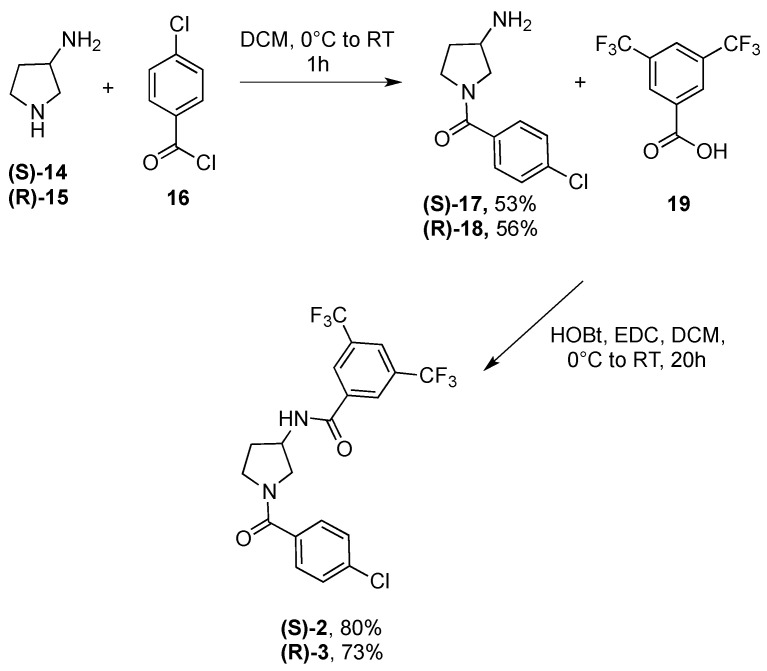
Synthesis of compounds **2** and **3**.

Scheme 1 reports the synthesis of compounds **2** and **3**. Specifically, enantiopure (S)- and (R)-3-aminopyrrolidines (**14** and **15**) were acylated, without requiring additional protecting groups, via a dropwise addition of 4-chlorobenzoyl chloride (**16**) in DCM at 0 °C. Intermediates **17** and **18**, obtained in **53** and **56%** yields, respectively, underwent a coupling reaction with 3,5-bis(trifluoromethyl)-benzoic acid **19**, to give compounds **2** and **3** in 80 and 73% yields, respectively.

**Scheme 2:**
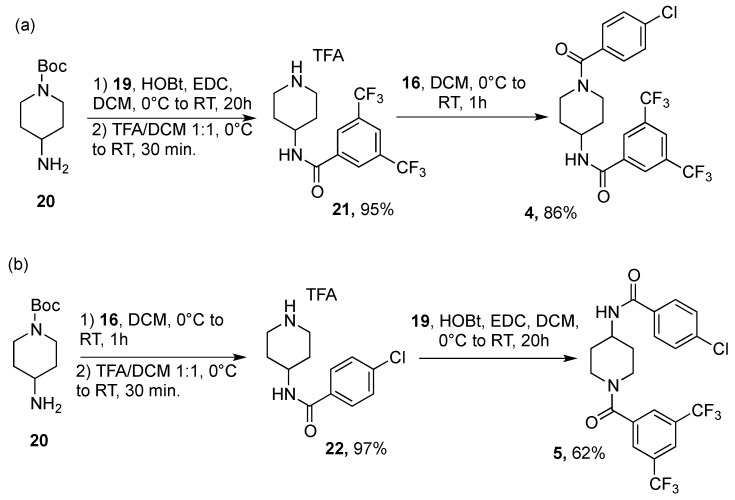
Synthesis of compound **4** (**a**) and **5** (**b**).

Compounds **4** and **5** were synthesized as depicted in Scheme 2 (**a** and **b**, respectively). Commercially available 4-amino-1-Boc-piperidine **20** was reacted with 3,5-bis(trifluoromethyl)-benzoic acid **19** to give intermediate **21** in 95% yield after two steps. The latter underwent acylation with **16** to give the desired derivative **4** in 86% yield. Similarly, compound **5** was synthesized via a three-steps sequence involving acylation with **16**, TFA promoted amine deprotection, and final coupling reaction with **19** (Scheme 2b).

Simultaneously, in the search for new chemical entities able to target MICAL2, we wondered if advanced synthetic tools, such as multicomponent reactions (MCRs) could be exploited. The application of MCRs to medicinal chemistry programs is nowadays considered an added value thanks to an innate atom economy, convergence, and the ability of creating in short times large libraries of drug-like compounds endowed with diversity and complexity [17,18].

**Scheme 3:**
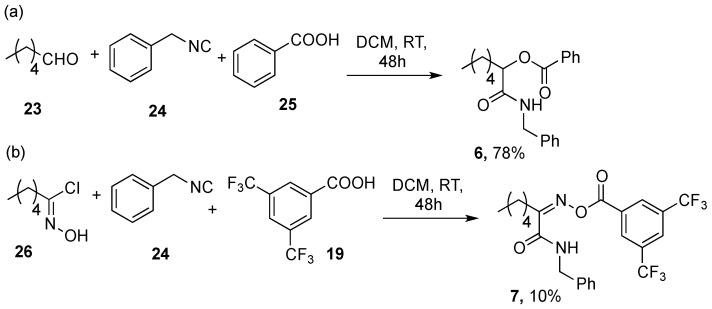
Synthesis of compounds **6** (**a**) and **7** (**b**).

To this end, compounds **6** and **7** were readily synthesized in just one reaction step via two different one-pot three-component reactions (3-CR): a Passerini 3-CR (for compound **6**, Scheme 3a), starting from *n*-hexanal **23**, benzyl isocyanide **24**, and benzoic acid **25** and a Passerini-like 3-CR (compound **7**, Scheme 3b) by combining *Z*-chlorooxime **26**, benzyl isocyanide **24**, and carboxylic acid **19** [19,20].

**Scheme 4:**
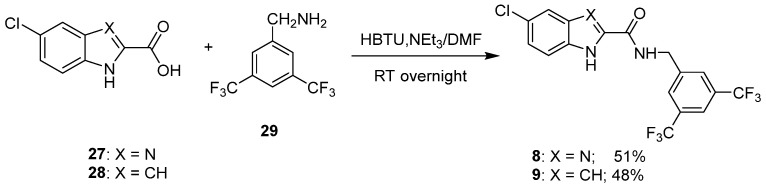
Synthesis of compounds **8** and **9**.

Scheme 4 reports the synthesis of compounds **8** and **9**. The direct coupling reaction of the appropriate commercially available carboxylic acid (5-chlorobenzimidazole-2-carboxylic acid **27** or 5-chloroindole-2-carboxylic acid **28**) with 3,5-bis(trifluoromethyl)benzylamine **29**, by carboxylate activation using *O*-benzotriazole-*N*,*N*,*N′*,*N′*-tetramethyluronium hexafluorophosphate (HBTU) in the presence of NEt_3_ in dry DMF at room temperature overnight, yielded the target compounds **8** and **9**, finally purified by recrystallization from ethanol/water.

**Scheme 5:**
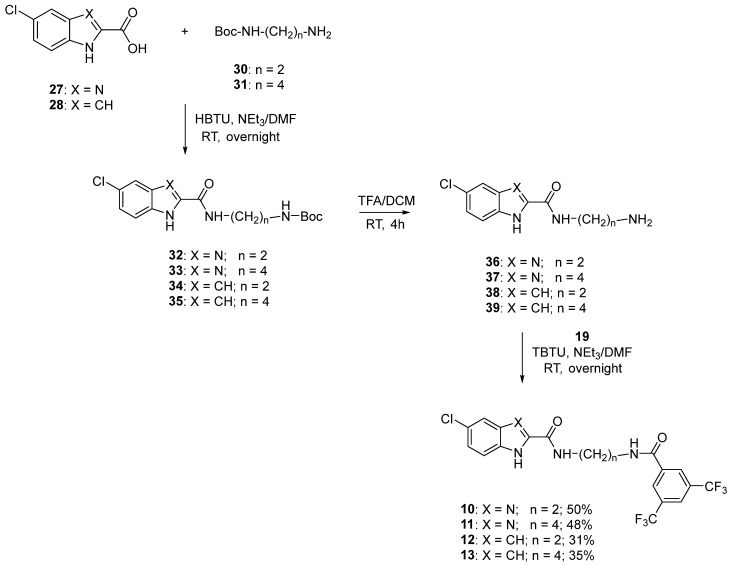
Synthesis of compounds **10**–**13**.

Target compounds **10**–**13** were prepared according to the experimental procedure outlined in Scheme 5. The first step consists of the condensation of the suitable carboxylic acid (5-chlorobenzimidazole-2-carboxylic acid **27** or 5-chloroindole-2-carboxylic acid **28**) with the appropriate *N*-boc-alkylenediamine (either *N*-boc-ethylenediamine **30** for **32** and **34** or *N*-boc-butylenediamine **31** for **33** and **35**) in anhydrous DMF, in the presence of HBTU and NEt_3_ at room temperature overnight. Subsequent Boc deprotection of **32**–**35** by treatment with trifluoroacetic acid for 4 h in dichloromethane furnished amines **36**–**39**, which after coupling reaction with 3,5-bis(trifluoromethyl)benzoic acid **19** in anhydrous DMF, using 2-(1*H*-benzotriazole-1-yl)-1,1,3,3-tetramethylaminium tetrafluoroborate (TBTU) as activating agent in the presence of DIPEA, provided the target compounds **10**–**13** finally purified by flash chromatography (for **10**) or by recrystallization from the appropriate solvent (for **11**–**13**).

### 2.2. Protein Expression

To obtain high levels of MICAL2 protein (Redox and Calponin homology domains), different constructs of the recombinant protein were expressed and purified. MICAL2_1-629_ was expressed as fusion protein containing a N-terminal glutathione-S-transferase (GST), Maltose-binding protein (MBP), NUS, GB1 tags that allow an easy purification by using the Gateway technology (Invitrogen). Unfortunately, despite the use of different strains of *Escherichia coli*, cell growth conditions, and Isopropil-β-D-1-tiogalattopiranoside (IPTG) concentrations, it was not possible to obtain appreciable levels of overexpression of MICAL2_1-629_ for biophysical assays. Since we were not able to overcome the difficulties arose in expressing the MICAL2 protein, and a full-length MICAL2 is not commercially available, we decided to directly test our compounds in cell-based assays.

### 2.3. Biological Assays

**Figure 2:**
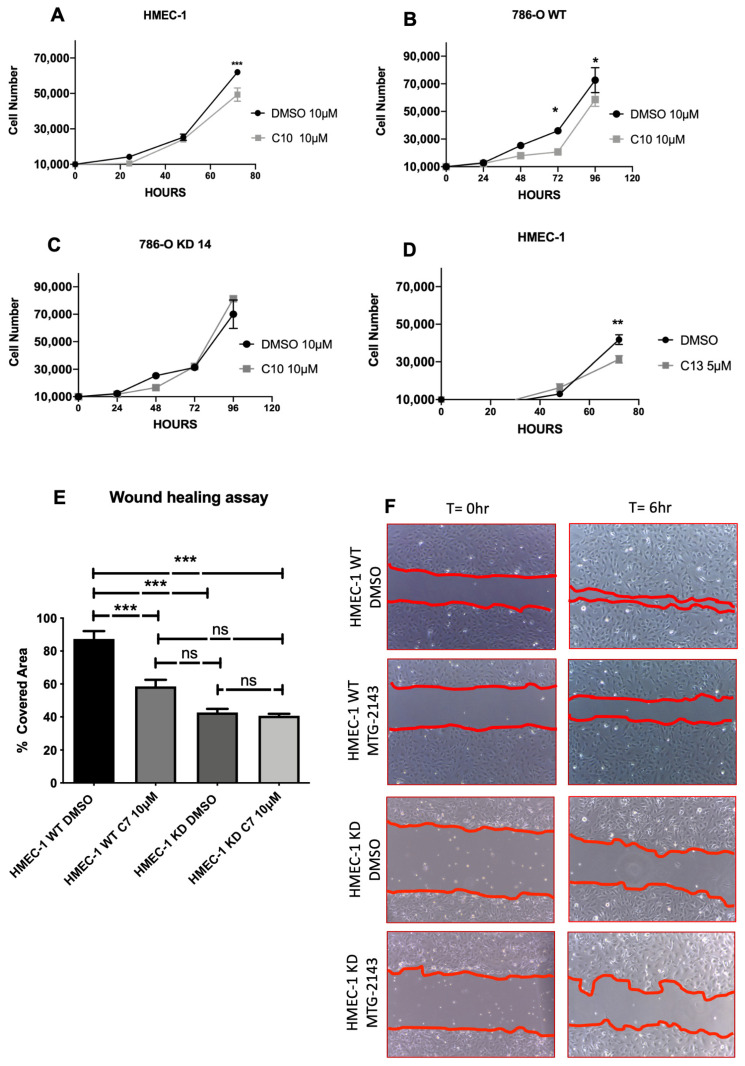
Results of cell biology assays performed with the most promising compounds. Cell viability assays, as in [16], showed that 10 μM compound **10** (**A**) and 5 μM compound **13** (**B**) exerted 35% and 25% reduction in live HMEC-1 cells at 72 h, respectively. Compound **10**, tested at 10 μM concentration on 786-O kidney cancer cells, produced 30% number reduction in parental cells at 96 h (**C**), whereas it did not exert any effect on the same cells in which MICAL2 was knocked-down (**D**), suggesting the inhibition effect might be mediated by the MICAL2 protein. In all graphs, dots represent cell number at time points. Cell numbers are expressed as mean ± SEM and were obtained from four biological replicas each performed with three technical replicas. Statistical analysis was performed with Two-way Anova test and Tukey’s Multiple Comparison post hoc test. * *p* ≤ 0.05, ** *p* ≤ 0.01, *** *p* ≤ 0.001. (**E**) Effect of compound **7** at 10 μM concentration on WHA with HMEC-1 ECs expressing MICAL2 and MICAL2-KD, performed after 48 h of exposure to drug. Scratch coverage was reduced of 40% in the test condition. In all graph bars, horizontal lines denote mean and SEM obtained from four independent biological replicas, each performed with three technical replicas. Statistical analysis was performed with One-way Anova test and Tukey’s Multiple Comparison post hoc test. *p*-value < 0.0001 (***). (**F**) Representative images of the results at six hours from scratch.

To test the capability of **2**–**13** to inhibit cell proliferation, viability assays were carried out incubating human dermal microvascular endothelial cells (HMEC-1) and renal cell adenocarcinoma cells (786-O) with each newly synthesized compound. Being cell motility, as well as cell proliferation, a critical feature of cancer progression and/or neoangiogenesis, also a WHA on HMEC-1 cells has been performed. All tested compounds did not show any relevant effect in these biological assays, with the exception of **10**, **13**, and **7**. Intriguingly, as shown in Figure 2A, after 72 h, compound **10** (10 µM) determined a significant reduction (35%) in vital HMEC-1 cells compared with the control, although it did not cause any effect on 2D motility in WHA (not shown), suggesting that compound **10** somehow specifically affects cell viability but not adhesion or motility. Similar results were obtained on 786-O cells, where a 30% reduction in parental cell number has been detected (Figure 2C). Importantly, **10** had no effect on MICAL2 knocked down 786-O cells (MICAL2-KD cells), suggesting the effect is somehow MICAL2 dependent (Figure 2D). Along the same lines, as shown in Figure 2B, challenging HMEC-1 cells for 72 h with compound **13** (5 µM), determined a significant reduction in vital cells (25%)*,* but similarly to **10** had no effect on 2D motility (not shown). Unlike compounds **10** and **13**, compound **7** (10 µM) elicited no effect on HMEC-1 cell number up to 72 h (not shown), but it caused 40% reduction in performance in WHA (Figure 2E,F). Of note, it had no effect on MICAL2-KD ECs (Figure 2E,F).

## 3. Molecular Modeling

**Figure 3:**
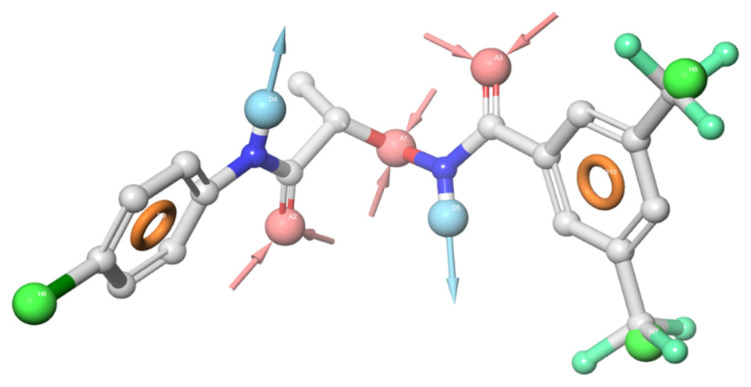
Ball and stick 3D representation of the lowest-energy minimum conformation of compound **1** (Cluster 4, S stereoisomer). Points used by Phase module of Schrödinger suite for the superposition with the newly synthesized compounds were highlighted.

To date, neither the X-ray structure of human MICAL-2 alone or in combination with inhibitors has been released, nor is the exact binding site of the only inhibitor known to date (compound **1**). Thus, a receptor-based approach is not feasible at the moment. However, starting from compound **1**, it has been possible to investigate a possible 3D superposition between **1** and each new analogue. First of all, in absence of the binding conformation of **1** into its target protein, a conformational analysis has been accomplished with the aid of Macromodel (Schrodinger package, Maestro Version 12.2.012, Release 2019-4). The resulting pool of 171 conformers was divided into 10 clusters on the base of the atomic RMSD and evaluated on the base of their potential energy (see Table 1 and Materials and Methods for details). Five clusters (1, 2, 8, 9 and 10) were characterized by a high average potential energy (≥21 kJ/mol) and/or contained conformers with one or both amide bonds in cis conformation, thus they were discarded. The other clusters included lower energy conformers with no geometry distortions. In particular, Cluster 4 was the most populated (47 conformers) and contained the lowest-energy minimum conformation which has been used for further analysis. Specifically, this conformer was processed with the Phase module of Schrödinger suite, that found ten points potentially important for the binding (see Figure 3): three hydrophobic groups (H, green spheres), three hydrogen bond receptor groups (A, pink spheres), two aromatic rings (R, orange circles), and two hydrogen bond donors (D, light blue spheres).

Then, in the attempt to find the best superposition between each newly synthesized ligand and compound **1**, the Phase module was used again. For each ligand, Phase program finds the best conformer to maximize the match with the chosen points of **1**.

**Figure 4:**
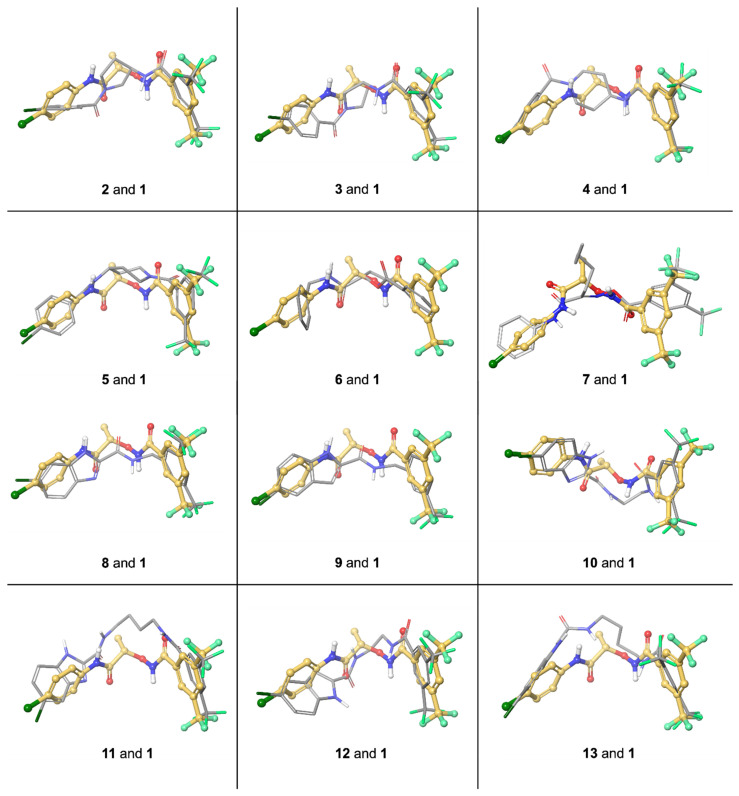
Best alignment of compounds **2**–**13** on the lowest energy minimum conformation of **1** (Cluster 4), as obtained with the Phase module.

Looking at this data and keeping in mind the in cell data, we can assert that substitution of the bridge CONHOCH(CH_3_)CONH) of compound **1**, with groups such as *N*-carboxypyrrolidine (**2** and **3**, Figure 1 and Figure 4) or *N*-carboxypiperidine (**4** and **5**, Figure 1 and Figure 4) completely abolishes the compounds activity. In case of **2** and **3**, this might be due to either to a different orientation of the 4-chlorophenyl ring that the novel bridge imposes either to its bulkiness with respect to the bridge of **1**, or to a loss of the intact amide group next the chlorophenyl moiety. The importance of these elements would be confirmed by compounds **4** and **5,** possessing a *N*-carboxypiperidine instead.

The most interesting series seems that of compounds **8**–**13** (although **8** and **9** have both linkers too short compared with **1**), where two ligands (**10** and **13**) were found to have activity on cell proliferation in both cell lines tested, whereas activity disappeared when a knockdown of MICAL2 was accomplished. Interestingly, differently from many other compounds here presented, the NH donor, next to the chlorophenyl ring, is conserved along with the adjacent aromatic moiety, and in the case of **10** and **11,** an acceptor mimicking the CO in the amide bond is also present (Figure 4).

Thus, although a full SARs rationalization is not feasible at the moment, due to the lack of any tests on isolated enzyme and any information on the MICAL2 exact site of binding, the work here reported clearly points out that the linker which connects the 3,5-bis(trifluoromethyl)phenyl with the 4-chlorophenyl plays not only the role of spacer, but probably it is important for the receptor interaction. Compound **10** would demonstrate than enlargement of the 4-chlorophenyl ring aromatic surface is possible if the adjacent donor/acceptor pattern is conserved. Synthesis of other compounds is necessary to add new pieces to the complex story of MICAL-2 inhibitors recognition and binding.

## 4. Discussion and Conclusions

We previously showed that MICAL2 is a new prometastatic gene, expressed in a variety of solid metastatic human cancer types [7] and it is expressed in ECs of neo-angiogenic capillaries irrorating solid tumors and in inflammation-related neo-angiogenesis [13]. Abating MICAL2 in cancer cells was enough to cause a strong phenotype of mesenchymal to epithelial transition, with marked loss of invasive properties. Reduction in MICAL2 in ECs impaired their angiogenic performance and released them from responding to VEGF, a most wanted target of therapies hitting unwanted neo-angiogenesis, which still call for new and more effective approaches [21].

Although significant findings stress a growing relevance of MICAL2 to human cancer, it is worth mentioning that only one small-molecule inhibitor of MICAL2, named CCG-1423 (**1**, Figure 1), exists at the moment. Herein, with the aim to enlarge the arsenal of pharmacological tools, starting from CCG-1423 molecular structure, a small library of structurally diverse ligands, retaining a variable level of similarity with **1**, was synthetized and biologically evaluated in human dermal microvascular endothelium cells (HMEC-1) and in renal cell adenocarcinoma (786-O) cells. Specifically, compound **10** gave, at 10 µM concentration, promising results in terms of reduction in cancer cell proliferation in both cell lines tested, whereas **7** showed an inhibitory effect on 2D cell motility. Furthermore, the null effect observed treating MICAL2-KD cells with either compound **10** or **7** suggests that the observed biological effects are mediated by MICAL2 protein, via a direct or indirect interaction with the protein. Indeed, the chemical similarity among **10**, **7** and **1** would suggest the first hypothesis. To add more complexity to the matter, the effects of **10** and **7** on cell function are not the same, although both typical of MICAL2 inhibition. Even if we cannot fully explain this observation as few information are available on MICAL2 multiple functions and on how they are regulated by small molecules binding, in principle it is possible that a biased signaling exists for structurally different compounds upon MICAL2 binding. This fascinating hypothesis will be surely the subject of further work. Besides the interesting biological activities of the novel compounds, this work provides the first structure–activity relationships (SARs) data of CCG-1423 **(1)** as MICAL2 inhibitor, thus paving the way for discovery of new MICAL2 inhibitors. Based on the previous promising results, and the availability of the synthetic protein for in vitro assays, it will be important to perform more biological assays with models of increasing complexity, from cell level up to dynamic co-cultures in vitro, and finally animal models. Increasing the number of pharmacological tools to inhibit the activity of MICAL2 will be useful in all contexts of unwanted cell proliferation and/or neoangiogenesis, at a hierarchy level downstream of VEGF, aiming at avoiding the collateral effects due to current antiangiogenic drugs.

## 5. Materials and Methods

### 5.1. Chemistry and General Methods

Commercially available reagents and solvents were used without further purification. When necessary, the reactions were performed in oven-dried glassware under a positive pressure of dry nitrogen. Uncorrected melting points were determined using a Reichert Kofler hot-stage apparatus (Reichert, Vienna, Austria). ^1^H and ^13^C APT NMR spectra were recorded on 400 MHz (see Supplementary Materials); ^13^C NMR spectra (Bruker, Massachusetts, US) were recorded on 100 MHz. Chemical shifts (δ) are reported in part per million (ppm) and coupling constants (J) are reported in hertz (Hz). High-resolution ESI-MS spectra were performed on a Thermo LTQ Orbitrap XL mass spectrometer (Thermo Fisher Scientific, San Jose, CA, USA). The spectra were recorded by infusion into the ESI source using MeOH as the solvent. Column chromatography was performed on silica gel (70–230 mesh ASTM) using the reported eluents. Thin layer chromatography (TLC) was carried out on 5 × 20 cm plates with a layer thickness of 0.25 mm (Silica gel 60 F_254_). When necessary, they were developed with ninhydrin solution.

#### 5.1.1. General Procedure for the Synthesis of Compounds **2**, **3**

Commercially available (*S*)- and (*R*)-3-aminopyrrolidines **14**,**15** (Merck KGaA, Darmstadt, Germany) (0.5 mmol) were dissolved in DCM (0.25 M), the solution was chilled at 0 °C and a cold 0.5 M solution of 4-chlorobenzoyl chloride **16** (0.9 equiv.) in DCM was added dropwise over 30 min. The reaction was stirred under a nitrogen atmosphere for additional 30 min. The crude mixture was then washed with a NaOH 5% aqueous solution, and the product was extracted with DCM (×4). The organic layers were dried over Na_2_SO_4_ and evaporated. Further purification via column chromatography afforded intermediates **17** and **18** in 53 and 56% yields, respectively. The latter were then dissolved in DCM (0.15 M), the solution was cooled at 0 °C and HOBt (1 equiv.), EDC HCl (0.9 equiv.), and 3,5-bis(trifluoromethyl)benzoic acid **19** (0.9 equiv.) were added. The reaction mixture was stirred at room temperature overnight, and washed with a 1N HCl solution (×3), NaHCO_3_ saturated solution (×3), and brine (×1). The crude mixture was further purified via column chromatography to afford **2** and **3** in 80 and 73% yield, respectively.

*(S)-N-(1-(4-Chlorobenzoyl)pyrrolidin-3-yl)-3,5-bis(trifluoromethyl)benzamide* (**2**). 1H NMR (400 MHz, CDCl3) δ 8.42 (s, 1H), 8.26 (s, 1H), 8.017.99–7.85 (m, 2H), 7.40–7.32 (m, 2H), 7.16 (m, 2H), 4.84–4.73 (m, 1H), 4.20–4.10 (m, 1H), 3.90–3.87 (m, 1H), 3.75–3.51 (m, 2H), 2.42–2.29 (m, 1H), 2.20–2.14 (m, 1H). HRMS (ESI) *m*/*z* Calcd for C_20_H_16_ClF_6_N_2_O_2+_: 465.0799; Found: 465.0799 [M+H]^+^.

*(R)-N-(1-(4-Chlorobenzoyl)pyrrolidin-3-yl)-3,5-bis(trifluoromethyl)benzamide* (**3**). 1H NMR (400 MHz, CDCl3) δ 8.40 (s, 1H), 8.25 (s, 1H), 7.978–7.87 (m, 1H), 7.75–7.68 (m, 1H), 7.43–7.33 (m, 2H), 7.19–7.17 (s, 2H), 4.8381–4.71 72 (m, 1H), 4.1615–4.09 11 (m, 1H), 3.8991–3.8386 (m, 1H), 3.7673–3.4849 (m, 2H), 2.4339–2.2831 (m, 1H), 2.1917–2.0204 (m, 1H). HRMS (ESI) *m*/*z* Calcd for C_20_H_16_ClF_6_N_2_O_2+_: 465.0799; Found: 465.0800 [M+H]^+^.

#### 5.1.2. Synthesis of Compound **4**

Commercially available 4-amino-1-Boc-pyrrolidine **20** (synthesized according to literature [22]) (0.5 mmol) was dissolved in CHCl_3_ (0.3 M); HOBt (1.2 equiv.), EDC HCl (1.1 equiv.), and 3,5-bis(trifluoromethyl)benzoic acid **19** (1 equiv.) were added, and the reaction was stirred at room temperature overnight. The crude mixture was washed with a citric acid solution (×2), NaHCO_3_ saturated solution (×2), and brine (×1). The crude mixture was further purified via column chromatography to afford intermediate **21** in 95% yield. The latter was dissolved in a 0.5 M 1:1 solution at 0 °C of TFA/DCM and stirred at room temperature for 30 min. Evaporation of the crude mixture gave deprotected **21** as TFA salt in quantitative yield. Intermediate **21** TFA was finally dissolved in DCM (0.2 M), cooled at 0 °C, and TEA (3 equiv.) and 4-chlorobenzoyl chloride **16** (1.5 equiv.) was added dropwise. The crude mixture was stirred under nitrogen for 2 h, washed with 1N HCl solution (×2), NaHCO_3_ saturated solution (×2), and brine (×1). The organic layer was dried under vacuum and purified via column chromatography to afford **4** in 86% yield.

*N-(1-(4-Chlorobenzoyl)piperidin-4-yl)-3,5-bis(trifluoromethyl)benzamide* (**4**)**.** ^1^H NMR (400 MHz, CDCl_3_) δ 8.32 (s, 2H), 7.90 (s, 1H), 7.81 (br d, -NH), 7.32 (d, *J* = 8.4 Hz, 2H), 7.25 (d, *J* = 8.4 Hz, 2H), 4.73–4.72 (m, 1H), 4.32–4.25 (m, 1H), 3.78–3.76 (m, 1H), 3.21–2.91 (m, 2H), 2.09–2.07 (m, 2H), 1.66–1.33 (m, 2H). HRMS (ESI) **m*/*z** Calcd for C_21_H_18_ClF_6_N_2_O_2_^+^: 479.0956; Found: 479.0969 [M+H]^+^.

#### 5.1.3. Synthesis of Compound **5**

Commercially available 4-amino-1-Boc-pyrrolidine **20** (synthesized according to literature [22]) (0.5 mmol) was dissolved in DCM (0.2 M), cooled at 0 °C, and TEA (3 equiv.) and 4-chlorobenzoyl chloride **16** (1.5 equiv.) was added dropwise. The crude mixture was stirred under nitrogen for 2 h, washed with a citric acid solution (×2), NaHCO_3_ saturated solution (×2), and brine (×1). The crude mixture was further purified via column chromatography to afford intermediate **22** in 97% yield. The latter was dissolved in a 0.5 M 1:1 solution at 0 °C of TFA/DCM and stirred at room temperature for 30 min. Evaporation of the crude mixture gave deprotected **22** as TFA salt in quantitative yield. Intermediate **22** TFA was finally dissolved in in CHCl_3_ (0.3 M); HOBt (1.2 equiv.), EDC HCl (1.1 equiv.), and 3,5-bis(trifluoromethyl)benzoic acid **19** (1 equiv.) were added, and the reaction was stirred at room temperature overnight. The reaction mixture was washed with 1N HCl solution (×2), NaHCO_3_ saturated solution (×2), and brine (×1). The organic layer was dried under vacuum and purified via column chromatography to afford **5** in 62% yield.

*N-(1-(3,5-bis(Trifluoromethyl)benzoyl)piperidin-4-yl)-4-chlorobenzamide* (**5**)**.** 1H NMR (400 MHz, CDCl3) δ 7.92 (s, 1H), 7.82 (s, 2H), 7.66 (d, J = 7.6 Hz, 2H), 7.35 (d, J = 7.6 Hz, 2H), 6.28 (br d, -NH), 3.63–3.59 (m, 1H), 3.22–2.97 (m, 2H), 2.11–2.01 (m, 2H), 1.57–1.39 (m, 2H); 13C NMR (400 MHz, CDCl3) δ 138.2, 137.4, 132.8, 131.9 (q, J = 33.5 Hz), 128.6, 128.3, 127.2 (d, J = 2.8 Hz), 123.2 (br q), 123.0 (q, J = 270.4 Hz), 47.1, 41.3, 31.3, 30.7, 22.3. HRMS (ESI) **m*/*z** Calcd for C_21_H_18_ClF_6_N_2_O_2+_: 479.0956; Found: 479.0957 [M+H]^+^.

#### 5.1.4. Synthesis of Compound **6**

Commercially available *n*-hexanal **23** (0.5 mmol) (Merck KGaA, Darmstadt, Germany), benzyl isocyanide **24** (1 equiv.) (Merck KGaA, Darmstadt, Germany), and benzoic acid **25** (1 equiv.) (Merck KGaA, Darmstadt, Germany) were one-pot mixed in DCM (0.5 M) and stirred at room temperature for 48 h. The crude mixture was purified via column chromatography to afford **6** in 78% yield.

*1-(Benzylamino)-1-oxoheptan-2-yl benzoate* (**6**)**.** ^1^H NMR (400 MHz, CDCl_3_) δ 8.05 (d, *J* = 7.6 Hz, 2H), 7.62–7.58 (m, 1H), 7.48–7.45 (m, 2H), 7.33–7.24 (m, 5H), 6.38–6.35 (m, -N*H*), 5.50–5.47 (m, 1H), 4.57–4.42 (m, 2H), 2.06–1.99 (m, 2H), 1.47–1.43 (m, 2H), 1.34–1.28 (m, 4H), 0.89–0.85 (m, 3H). HRMS (ESI) **m*/*z** Calcd for C_21_H_26_NO_3_^+^: 340.1908; Found: 340.1903 [M+H]^+^.

#### 5.1.5. Synthesis of Compound **7**

(*Z*)-*N*-hydroxyhexanimidoyl chloride **26** (1 mmol), benzyl isocyanide **24** (1 equiv.), 3,5-bis(trifluoromethyl)benzoic acid **19** (1 equiv.), and TEA (2 equiv.) were one-pot mixed in DMF (1 M) and stirred at room temperature overnight. The crude mixture was washed with brine (×3), evaporated under vacuum, and purified via column chromatography to afford **7** in 10% yield.

*(Z)-N-Benzyl-2-(((3,5-bis(trifluoromethyl)benzoyl)oxy)imino)heptanamide* (**7**)**.** ^1^H NMR (400 MHz, CDCl_3_) δ 8.37 (s, 2H), 8.10 (s, 1H), 7.22–7.18 (m, 5H), 6.16 (br t, -N*H*), 4.57 (d, *J* = 5.6 Hz, 1H), 2.68–2.64 (m, 2H), 1.68–1.63 (m, 2H), 1.39–1.31 (m, 4H), 0.89 (t, *J* = 6.8 Hz, 3H). HRMS (ESI) **m*/*z** Calcd for C_23_H_22_F_6_N_2_NaO_3_^+^: 511.1432; Found: 511.1430 [M+Na]^+^.

#### 5.1.6. General Procedure for the Synthesis of Compounds **8** and **9**

To a solution of the appropriate commercially available carboxylic acid, 5-chloro-1*H*-benzo[*d*]imidazole-2-carboxylic acid **27** or 5-chloro-1*H*-indole-2-carboxylic acid **28** (1.02 mmol) in dry DMF (1.5 mL), HBTU (426 mg, 1.12 mmol), NEt_3_ (0.16 mL, 1.12 mmol) and 3,5-bis(trifluoromethyl)benzylamine **29** (272 mg, 1.12 mmol) were added under a nitrogen atmosphere. The reaction was stirred at room temperature overnight and then concentrated in vacuo. The residue was taken up with 0 °C water (15 mL) and the formed precipitate was collected by filtration and purified by recrystallization from ethanol/water to afford compounds **8** and **9** in 51 and 48% yield, respectively.

*N-(3,5-bis(Trifluoromethyl)benzyl)-5-chloro-1H-benzo[d]imidazole-2-carboxamide* (**8**): m.p. 202–204 °C; ^1^H NMR (400 Hz, DMSO-d_6_, δ ppm) 13.51 (bs, 1H), 9.82 (t, 1H, *J* = 5.8 Hz), 8.08 (s, 2H), 8.02 (s, 1H), 7.80–7.75 (m, 1H), 7.56–7.54 (m, 1H), 7.36–7.30 (m, 1H), 4.68 (d, 2H, *J* = 6.0 Hz); ^13^C NMR (100 Hz, DMSO-d_6_, δ ppm) 159.30, 143.06, 130.62 (q, *J*_CF_ = 33.0 Hz), 130.46, 128.88, 123.82 (q, *J*_CF_ = 271.0 Hz), 121.30, 121.27, 121.23, 42.18; HRMS (ESI) *m*/*z* Calcd for C_17_H_11_ClF_6_N_3_O^+^: 422.0495; Found: 422.0501 [M+H]^+^.

*N-(3,5-bis(Trifluoromethyl)benzyl)-5-chloro-1H-indole-2-carboxamide* (**9**): m.p. 239–241 °C; ^1^H NMR (400 Hz, DMSO-d_6_, δ ppm) 11.89 (s, 1H), 9.30 (t, 1H, *J* = 6.0 Hz), 8.05 (s, 2H), 8.03 (s, 1H), 7.74 (d, 1H, *J* = 2.0 Hz), 7.43 (d, 1H, *J* = 8.8 Hz), 7.20 (dd, 1H, *J* = 2.0 Hz, *J* = 8.8 Hz), 7.18–7.17 (m, 1H), 4.69 (d, 2H, *J* = 6.0 Hz); ^13^C NMR (100 Hz, DMSO-d_6_, δ ppm) 161.59, 143.60, 135.41, 133.05, 130.51, 130.67 (q, *J*_CF_ = 32.0 Hz), 128.67, 128.55, 123.83 (q, *J*_CF_ = 271.0 Hz), 124.78, 124.11, 121.16, 114.40, 102.98, 42.09; HRMS (ESI) **m*/*z** Calcd for C_18_H_12_ClF_6_N_2_O^+^: 421.0542; Found: 421.0551 [M+H]^+^.

#### 5.1.7. General Procedure for the Synthesis of Compounds **10**–**13**

To a solution of 3,5-bis(trifluoromethyl)carboxylic acid **19** (290 mg, 1.12 mmol) in dry DMF (4.0 mL), TBTU (481 mg, 1.50 mmol), DIPEA (0.39 mL, 2.24 mmol) and the appropriate derivatives **36**–**39** (1.12 mmol) were added under a nitrogen atmosphere. The reaction was stirred at room temperature overnight and then concentrated in vacuo. The residue was taken up with 0 °C water (20 mL) and the formed precipitate was collected by filtration and purified by column chromatography (for **10**) or recrystallization from the appropriate solvent (for **11–13**) to afford title compounds **10**–**13** in 31–50% yield.

*N-(2-(3,5-bis(trifluoromethyl)benzamido)ethyl)-5-chloro-1H-benzo[d]imidazole-2-carboxamide* (**10**) m.p. 251–253 °C; 1H NMR (400 Hz, DMSO-d6, δ ppm) 13.45 (bs, 1H), 9.19 (t, 1H, J = 6.0 Hz), 9.12 (t, 1H, J = 5.6 Hz), 8.49 (s, 2H), 8.33 (s, 1H), 7.65–7.61 (m, 2H), 7.31 (dd, 1H, J = 2.0 Hz, J = 8.4 Hz), 3.53 (s, 4H); 13C NMR (100 Hz, DMSO-d6, δ ppm) 164.01, 159.33, 147.61, 137.24, 130.87 (q, JCF = 33.0 Hz), 130.71, 128.55, 125.26, 123.60 (q, JCF = 271.0 Hz), 124.08, 38.94; HRMS (ESI) *m*/*z* Calcd for C_19_H_14_ClF_6_N_4_O_2+_: 479.0704; Found: 479.0700.[M+H]^+^.

*N-(2-(3,5-bis(Trifluoromethyl)benzamido)butyl)-5-chloro-1H-benzo[d]imidazole-2-carboxamide* (**11**)**:** m.p. 254–256 °C (ethanol/water); ^1^H NMR (400 Hz, DMSO-d_6_, δ ppm) 13.40 (bs, 1H), 9.06 (t, 1H, *J* = 6.0 Hz), 8.98 (t, 1H, *J* = 5.6 Hz), 8.50 (s, 2H), 8.32 (s, 1H), 7.76–7.71 (m, 1H), 7.54–7.50 (m, 1H), 7.34–7.28 (m, 1H), 3.40–3.36 (m, 4H), 1.63–1.60 (m, 4H); ^13^C NMR (100 Hz, DMSO-d_6_, δ ppm) 163.53, 158.81, 137.13, 131.06, 130.89 (q, *J*_CF_ = 33.0 Hz), 128.45, 125.21, 123.60 (q, *J*_CF_ = 271.0 Hz), 119.54, 114.42, 112.56, 38.98, 27.00, 26.78. HRMS (ESI) *m*/*z* Calcd for C_21_H_18_ClF_6_N_4_O_2_^+^: 507.1022; Found: 507.1034 [M+H]^+^.

*N-(2-(3,5-bis(Trifluoromethyl)benzamido)ethyl)-5-chloro-1H-indole-2-carboxamide* (**12**)**:** m.p. 270–272 °C (toluene); ^1^H NMR (400 Hz, DMSO-d_6_, δ ppm) 11.83 (s, 1H), 9.16 (t, 1H, *J* = 5.6 Hz), 8.76 (t, 1H, *J* = 5.6 Hz), 8.51 (s, 2H), 8.34 (s, 1H), 7.70 (d, 1H, *J* = 1.6 Hz), 7.42 (d, 1H, *J* = 8.4 Hz), 7.17 (dd, 1H, *J* = 2.2 Hz, *J* = 8.6 Hz), 7.09 (s, 1H), 3.51 (bs, 4H); ^13^C NMR (100 Hz, DMSO-d_6_, δ ppm) 164.02, 161.47, 137.16, 135.26, 133.67, 130.91 (q, *J*_CF_ = 33.0 Hz), 129.36, 128.66, 128.57, 124.65, 123.81, 123.60 (q, *J*_CF_ = 271.0 Hz), 121.00, 114.34, 102.52, 38.81; HRMS (ESI) **m*/*z** Calcd for C_20_H_15_ClF_6_N_3_O_2_^+^: 478.0757; Found: 478.0763 [M+H]^+^.

*N-(4-(3,5-bis(Trifluoromethyl)benzamido)butyl)-5-chloro-1H-indole-2-carboxamide* (**13**)**:** m.p. 267–269 °C (toluene); ^1^H NMR (400 Hz, DMSO-d_6_, δ ppm) 11.76 (s, 1H), 9.00 (t, 1H, *J* = 5.6 Hz), 8.57 (t, 1H, *J* = 5.6 Hz), 8.51 (s, 2H), 8.32 (s, 1H), 7.68 (d, 1H, *J* = 2.0 Hz), 7.42 (d, 1H, *J* = 8.8 Hz), 7.17 (dd, 1H, *J* = 2.2 Hz, *J* = 8.6 Hz), 7.09 (d, 1H, *J* = 1.6 Hz), 3.37–3.35 (m, 4H), 1.63–1.60 (m, 4H); ^13^C NMR (100 Hz, DMSO-d_6_, δ ppm) 163.54, 161.11, 137.11, 135.20, 133.84, 130.91 (q, *J*_CF_ = 34.0 Hz), 128.60, 128.46, 124.60, 123.71, 123.61 (q, *J*_CF_ = 272.0 Hz), 120.94, 114.31, 102.25, 38.95, 27.19, 26.88; HRMS (ESI) **m*/*z** Calcd for C_22_H_19_ClF_6_N_3_O_2_^+^: 506.1064; Found: 506.1078 [M+H]^+^.

#### 5.1.8. General Procedure for the Synthesis of Compounds **32**–**35**

To a solution of the appropriate commercially available carboxylic acid, 5-chloro-1*H*-benzo[*d*]imidazole-2-carboxylic acid **27** or 5-chloro-1*H*-indole-2-carboxylic acid **28** (1.02 mmol) in dry DMF (1.5 mL), HBTU (426 mg, 1.12 mmol), NEt_3_ (0.16 mL, 1.12 mmol), and the appropriate *N*-Boc-alkylenediamine (*N*-Boc-ethylenediamine **30** for **32** and **34**, *N*-Boc-butylenediamine **31** for **33** and **35**, 1.12 mmol), were added under a nitrogen atmosphere. The reaction was stirred at room temperature overnight and then concentrated in vacuo. The residue was taken up with 0 °C water (15 mL), and the formed precipitate was collected by filtration and purified by recrystallization from the appropriate solvent to afford compounds **32**–**35** in 35–42% yield.

*tert-Butyl (2-(5-chloro-1H-benzo[d]imidazole-2-carboxamido)ethyl)carbamate* (**32**)**:** m.p. 194–196 °C (ethanol/water); ^1^H NMR (400 Hz, DMSO-d_6_, δ ppm) 13.44 (bs, 1H), 8.99 (t, 1H, *J* = 5.6 Hz), 7.75–7.52 (m, 2H), 7.34–7.31 (m, 1H), 6.95 (t, 1H, *J* = 5.6 Hz), 3.37–3.34 (m, 2H), 3.14–3.12 (m, 2H), 1.37 (s, 9H).

*tert-Butyl (2-(5-chloro-1H-benzo[d]imidazole-2-carboxamido)butyl)carbamate* (**33**): m.p. 184–186 °C (ethanol/water); ^1^H NMR (400 Hz, DMSO-d_6_, δ ppm) 13.41 (bs, 1H), 9.02 (t, 1H, *J* = 6.0 Hz), 7.79–7.59 (m, 2H), 7.33–7.31 (m, 1H), 6.81 (t, 1H, *J* = 5.0 Hz), 3.31–3.26 (m, 2H), 2.95–2.91 (m, 2H), 1.56–1.49 (m, 2H), 1.44–1.36 (m, 2H), 1.37 (s, 9H).

*tert-Butyl (2-(5-chloro-1H-indole-2-carboxamido)ethyl)carbamate* (**34**)**:** m.p. 218–220 °C (toluene); ^1^H NMR (400 Hz, DMSO-d_6_, δ ppm) 11.80 (s, 1H), 8.57 (t, 1H, *J* = 5.6 Hz), 7.70 (d, 1H, *J* = 2.0 Hz), 7.42 (d, 1H, *J* = 8.8 Hz), 7.17 (dd, 1H, *J* = 2.0 Hz, *J* = 8.8 Hz), 7.08 (s, 1H), 6.94 (t, 1H, *J* = 5.6 Hz), 3.32–3.29 (m, 2H), 3.14–3.09 (m, 2H), 1.38 (s, 9H).

*tert-Butyl (2-(5-chloro-1H-indole-2-carboxamido)butyl)carbamate* (**35**): m.p. 200–202 °C (toluene); ^1^H NMR (400 Hz, DMSO-d_6_, δ ppm) 11.76 (s, 1H), 8.54 (t, 1H, *J* = 2.0 Hz), 7.69 (d, 1H, *J* = 2.0 Hz), 7.42 (d, 1H, *J* = 8.4 Hz), 7.17 (dd, 1H, *J* = 2.0 Hz, *J* = 8.8 Hz), 7.09 (d, 1H, *J* = 1.6 Hz), 6.83 (t, 1H, *J* = 5.6 Hz), 3.29–3.25 (m, 2H), 2.97–2.92 (m, 2H), 1.52–1.47 (m, 2H), 1.45–1.40 (m, 2H), 1.37 (s, 9H).

#### 5.1.9. General Procedure for the Synthesis of Compounds **36**–**39**

A stirred solution of the appropriate compound **32**–**35** (0.85 mmol) in dry CH_2_Cl_2_ (4 mL) was added dropwise with TFA (0.8 mL). After stirring at room temperature for 3 h, the reaction mixture was concentrated in vacuo. The solid residue was taken up with water, cooled in an ice bath, treated with 3M NaOH (0.8 mL), and extracted with CH_2_Cl_2_ (2 × 20 mL). The combined organic phases were washed with brine (6 mL), dried (Na_2_SO_4_), and concentrated in vacuo to obtain **36**–**39** in 59–100% yield with satisfactory purity degree.

*N-(2-Aminoethyl)-5-chloro-1H-benzo[d]imidazole-2-carboxamide* (**36**): oil; ^1^H NMR (400 Hz, DMSO-d_6_, δ ppm) 9.12 (t, 1H, J = 5.6 Hz), 8.45 (bs, 2H), 7.68–7.64 (m, 2H), 7.33 (dd, 1H, *J* = 2.0 Hz, *J* = 8.4 Hz), 3.52–3.48 (m, 2H), 2.95 (t, 2H, *J* = 6.2 Hz).

*N-(2-Aminobutyl)-5-chloro-1H-benzo[d]imidazole-2-carboxamide* (**37**): m.p. 214–216 °C; ^1^H NMR (DMSO-d_6_, δ ppm) 9.09 (t, 1H, *J* = 5.8 Hz), 7.66–7.63 (m, 2H), 7.33 (dd, 1H, *J* = 2.0 Hz, *J* = 8.8 Hz), 3.34–3.31 (m, 2H), 2.82 (t, 2H, *J* = 7.2 Hz), 1.60–1.58 (m, 4H).

*N-(2-Aminoethyl)-5-chloro-1H-indole-2-carboxamide* (**38**): m.p. 200–202 °C; ^1^H NMR (400 Hz, DMSO-d_6_, δ ppm) 8.60 (t, 1H, *J* = 5.8 Hz), 7.69 (d, 1H, *J* = 2.0 Hz), 7.43 (d, 1H, *J* = 8.8 Hz), 7.18 (dd, 1H, *J* = 2.0 Hz, *J* = 8.4 Hz), 7.11 (s, 1H), 3.40–3.36 (m, 2H), 2.82 (t, 2H, *J* = 6.6 Hz).

*N-(4-Aminobutyl)-5-chloro-1H-indole-2-carboxamide* (**39**): m.p. 210–212 °C; ^1^H NMR (400 Hz, DMSO-d_6_, δ ppm) 8.61 (t, 1H, *J* = 5.6 Hz), 7.69 (d, 1H, *J* = 2.0 Hz), 7.42 (d, 1H, *J* = 8.4 Hz), 7.17 (dd, 1H, *J* = 2.0 Hz, *J* = 8.8 Hz), 7.09 (s, 1H), 3.31–3.26 (m, 2H), 2.62 (t, 2H, *J* = 6.8 Hz), 1.59–1.50 (m, 2H), 1.47–1.40 (m, 2H).

### 5.2. Cell Lines, Culture Procedure

HMEC-1 (CDC, Atlanta, GA, USA), is a line of human microvascular ECs of dermal origin [23]. HMEC-1 cells were cultured as in [13], seeded on plastic (Sarstedt, Numbrect, Germany) coated with 2% gelatin to improve cell adhesion, in MCDB131 medium supplemented with 10% Fetal Bovine Serum (FBS, Gibco, Thermo Fisher Scientific, Waltham, MA, USA), hydrocortisone (1 μg/mL), EGF (10 ng/mL), 1 mM sodium bicarbonate, Penicillin (1 U/mL), Streptomycin (1 μg/mL), HEPES (20 mM).

786-O (ATCC CRL-1932, ATCC-LGC Standards, Eu, [24]), renal cell adenocarcinoma, and 786-O MICAL2 knock-down [7] were kept in RPMI-1640 with 10% heat-inactivated FBS (Gibco, Thermo Fisher Scientific, Waltham, MA, USA), 10 mM HEPES, 1 mM Na pyruvate, 2 mM glutamine. Cells were routinely tested for mycoplasma contamination with DAPI staining or MycoAlert Mycoplasma Detection Kit (Lonza, Allendale, NJ, USA).

All media and supplements were from Sigma-Aldrich (Saint Louis, MO, USA), unless specified differently. Cell cultures were incubated at 37 °C in 5% CO_2_, 80% humidity.

#### 5.2.1. Manual Counting

All compounds were resuspended in Dimethyl sulfoxide (DMSO) at the final concentration of 11 mM. 10,000 cells were seeded in 24-well plates (Sarstedt, Numbrect, Germany). Three hours from seeding, the compound of interest was added, at the final concentration of 5 or 10 μM, respectively. Control plates received 0.045% or 0.09% vol/vol DMSO. Cells were manually counted with Trypan blue exclusion assay as in [16] after 24, 48, 72, and 96 h from drug administration.

Each test was repeated at least three times, each time with three technical replicas, at 24, 48, 72, and 96 h after seeding.

#### 5.2.2. 2D Migration Assay (Wound Healing Assay, WHA)

The WHA, also known as the scratch assay, is an established two-dimensional (2D) technique that can be used to investigate collective migration and wound healing in vitro. This method was one of the first to be developed for the study of cell migration and measures the rate at which cells, in a cell monolayer, migrate to fill a cell-free gap [15]. It is a multistep procedure involving (1) growing a cell monolayer to confluence in a multiwell assay plate; (2) creating a “wound”, a cell-free area in the monolayer, into which cells can subsequently migrate; (3) monitoring the recolonization of the cell-free gap to quantify cell motility. We seeded 200,000 cells per well, in 12-well plates (Sarstedt, Numbrect, Germany). Three hours from seeding, the drug of interest, or the vehicle, was added. A total of 48 h later, the scratch was performed, and fresh medium was added (T = 0 h). Each test was repeated at least three times, each time with three technical replicas; photos were taken at 0 and 6 h after the scratch, with Leica DM IL microscope; magnification: 10X. Image analysis was performed with ImageJ suite (NIH, Bethesda, MD, USA). The strategy to quantify wound closure is based on distinguishing cells from the background and measuring the change in the recolonized area. To quantify wound closure over time, images captured at the same time point were processed with WimScatch image analyzer Platform software (Wimasis Image Analysis, Cordoba, Spain).

### 5.3. Statistical Analysis

Data of WHA are indicated as mean ± SEM and analyzed with One-way Anova test and Tukey’s multiple comparison post hoc test. Data of viability assays were obtained from four independent experiments (each performed with three technical replicas). They are expressed as mean ± SEM and analyzed with a Two-way Anova test and Tukey’s multiple comparison post hoc test. Always, difference between means was judged statistically significant for *p* ≤ 0.05. All statistical procedures were performed using GraphPad (GraphPad Software, San Diego, CA, USA).

### 5.4. Molecular Modeling

Three-dimensional structures of all the molecules used in this study were built in Maestro 12.2 (Schrödinger, LLC, New York, NY, USA).

As for the conformational search of compound **1**, the ConfGen Module in MacroModel 12.6 was used with the OPLS_2005 force field, GB/SA water, and no cutoff for nonbonded interactions. Molecular energy minimizations were performed using the PRCG method with 2500 maximum iterations and 0.05 gradient convergence threshold. The conformational searches were carried out by application of the MCMM torsional sampling method, performing automatic setup with 21 kJ/mol in the energy window for saving structure and a 0.5 Å cutoff distance for redundant conformers. The output consisted in 171 conformers that were clustered with the Conformer Cluster module in MacroModel 12.6 on the base of atomic RMSD, retaining the mirror-image conformers and using the Average linkage method.

Pharmacophore feature sites of each selected conformer of compound **1** (from Clusters 3, 4, 5, 6 and 7) were assigned with the aid of Phase 4.8 using a set of features defined in Phase as hydrogen-bond acceptor (A), hydrogen-bond donor (D), hydrophobic group (H), negatively charged group (N), positively charged group (P), and aromatic ring (R). For each conformer one hypothesis with ten points was generated. Then, compounds **2**–**13** were processed with Phase against the five hypotheses. Screening molecules were required to match a minimum of four out of ten sites. The distance matching tolerance was set to 2.0 Å as a balance between stringent and loose-fitting matching alignment. Conformational sampling was automatically performed on all the molecules using the ConfGen search algorithm within Phase, with the automatic identification and alignment of pharmacophoric sites for each conformer on the reference compound.

## Figures and Tables

**Table 1:** Number of conformers and average energy of each conformational analysis cluster.

Cluster	1	2	3	4	5	6	7	8	9	10
N° conformers	15	15	15	47	21	9	30	2	9	8
Average Energy in kJ/mol	22.7	22.53	20.7	17.22	16.191	15.95	20.99	24.91	20.9	20.71

## Supplementary Materials

The following are available online. ^1^H spectra of representative compounds.
