# Supplementary material for: Enriching the Arsenal of Pharmacological Tools against MICAL2
Source: Molecules. 2021 Dec 11;26(24):7519. doi: 10.3390/molecules26247519 (PMC8709466; doi:10.3390/molecules26247519)
Supplement: Supplementary file 1 [file molecules-26-07519-s001.zip › molecules-1441595-supplementary.pdf]

SUPPORTING INFORMATION

Enriching the Arsenal of Pharmacological Tools against MICAL2

Ivana Barravecchia [1,2§], Elisabetta Barresi [2§], Camilla Russo[3§], Francesca Scebba [1], Chiara De Cesari[1], Valerio Mignucci[1], Davide De Luca[1], Silvia Salerno[2], Valeria La Pietra[3], Mariateresa Giustiniano[3], Sveva Pelliccia[3], Diego Brancaccio[3], Greta Donati[3], Federico Da Settimo[2], Sabrina Taliani[2] *, Debora Angeloni[1] *, Luciana Marinelli[3] *

[1] Institute of Life Sciences, Scuola Superiore Sant'Anna, Via G. Moruzzi, 1, 56124 Pisa, Italy.

[2] Department of Pharmacy, University of Pisa, Via Bonanno 6, 56126 Pisa, Italy.

[3] Dipartimento di Farmacia, Università degli Studi di Napoli "Federico II", Via D. Montesano 49, 80131 Naples, Italy.

[§] These authors equally contributed to the work.

Contents:

- Copies of ^{1}H spectra of representative compounds…………………...……….........S3

^{1}H NMR

400 MHz, $CDCl_3$

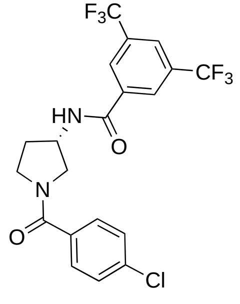

2

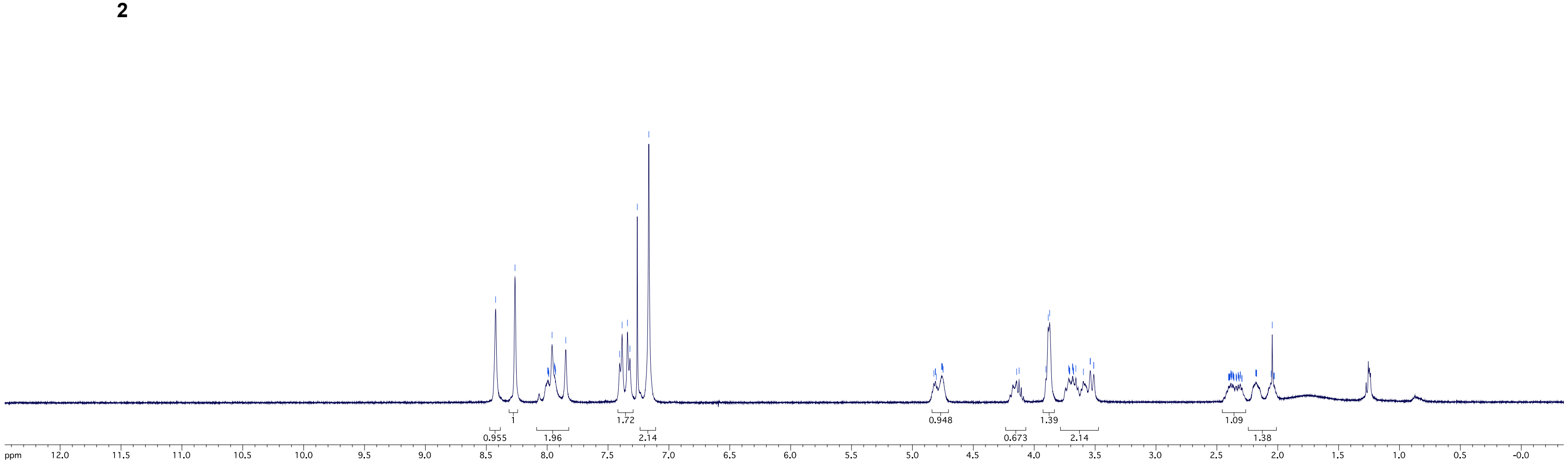

^{1}H NMR
400 MHz, $CDCl_3$

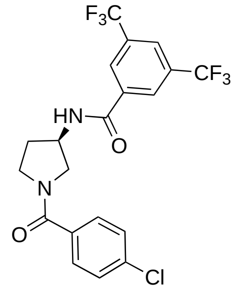

3

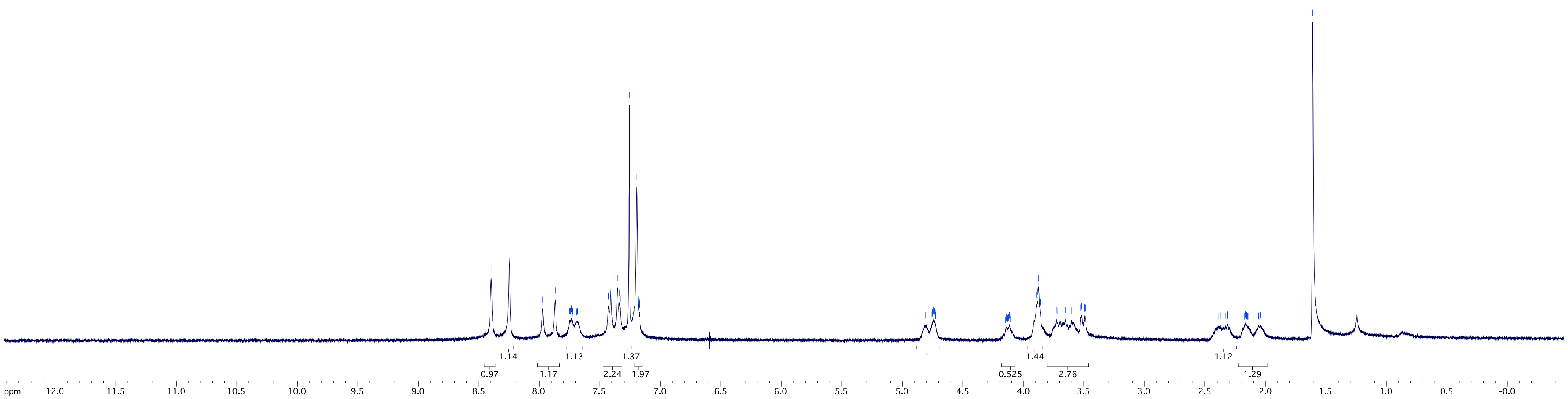

^{1}H NMR
400 MHz, $CDCl_3$

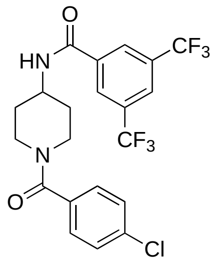

4

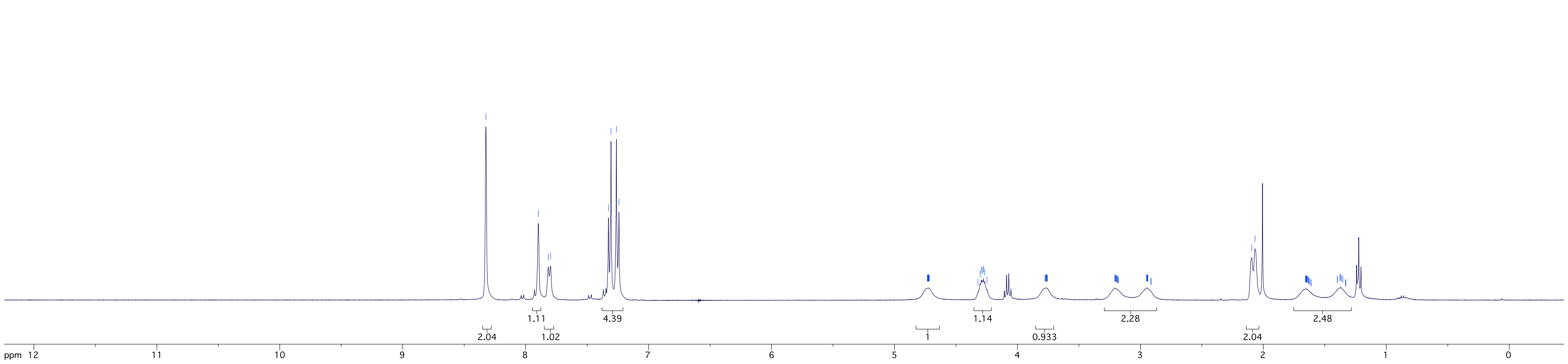

^{1}H NMR
400 MHz, $CDCl_3$

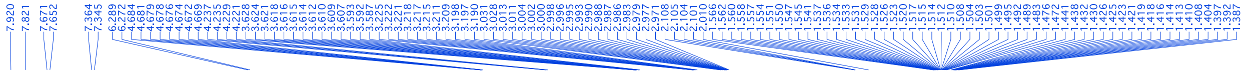

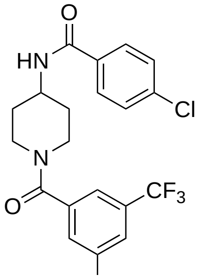

5

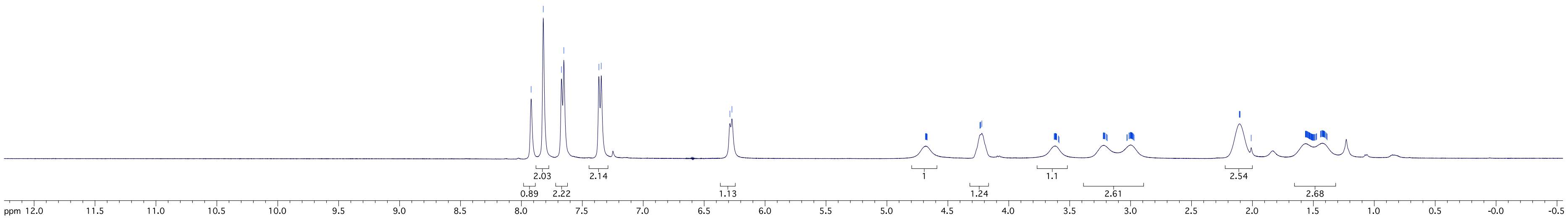

^{1}H NMR
400 MHz, $CDCl_3$

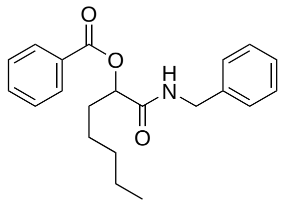

6

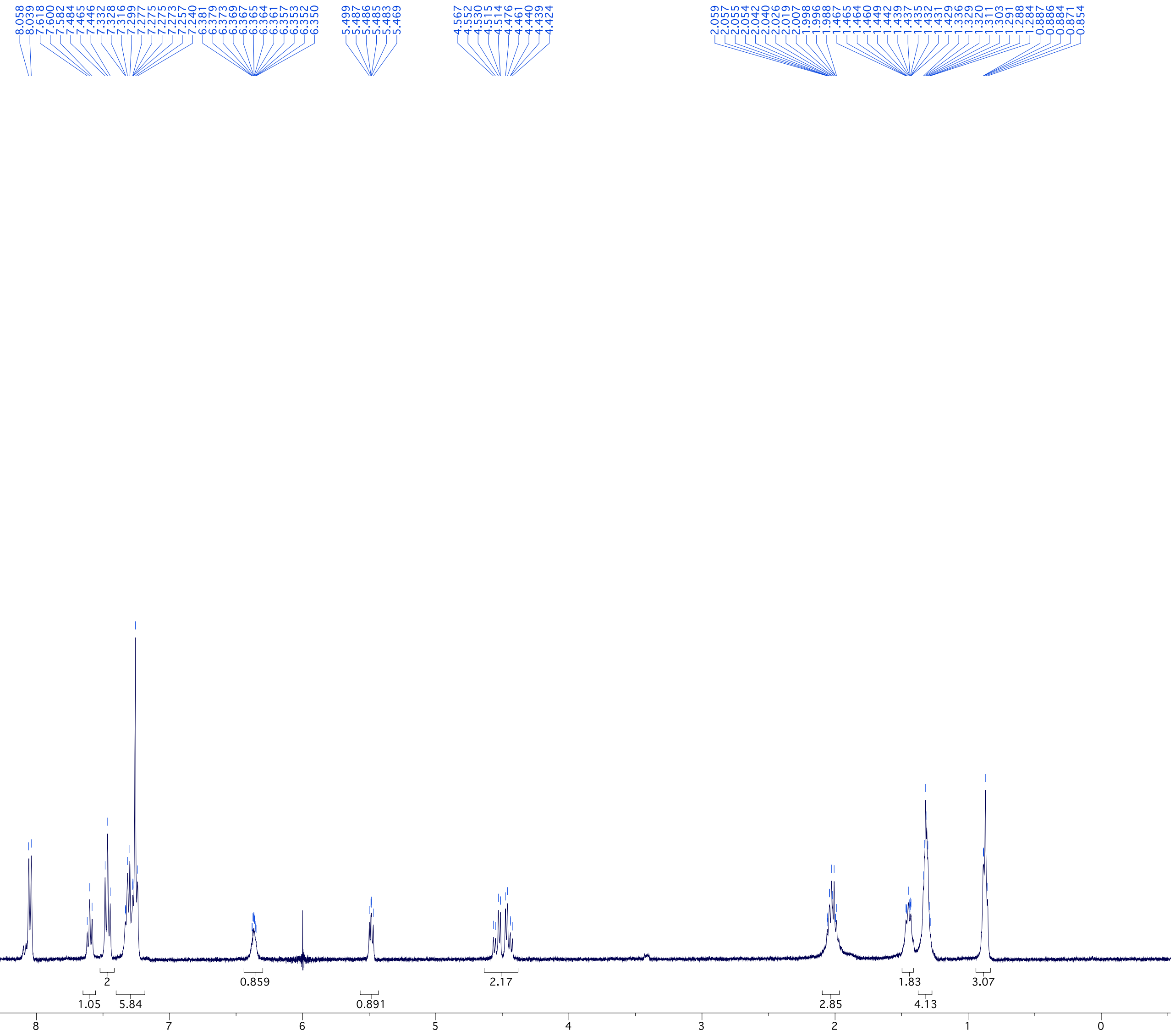

^{1}H NMR
400 MHz, $CDCl_3$

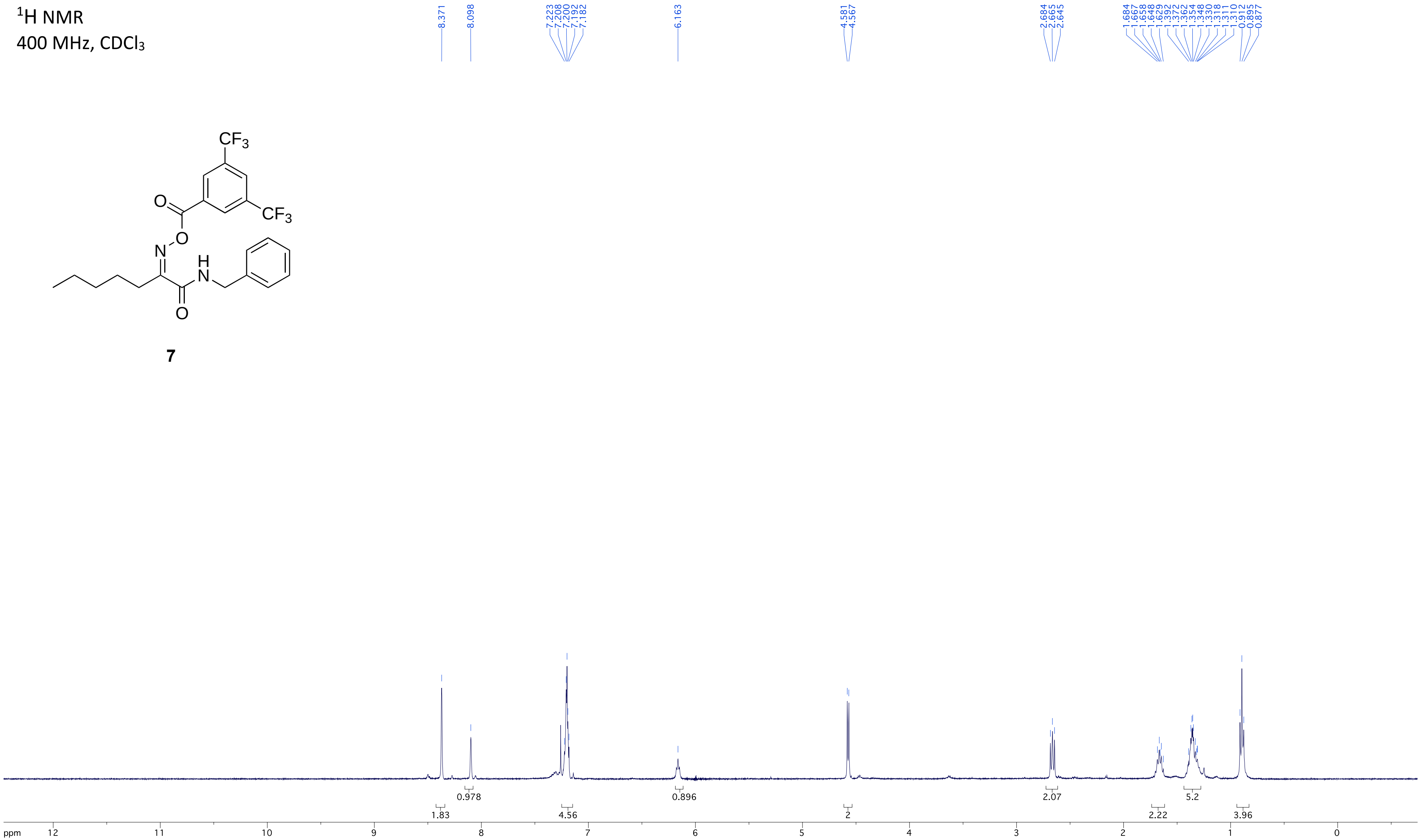

7

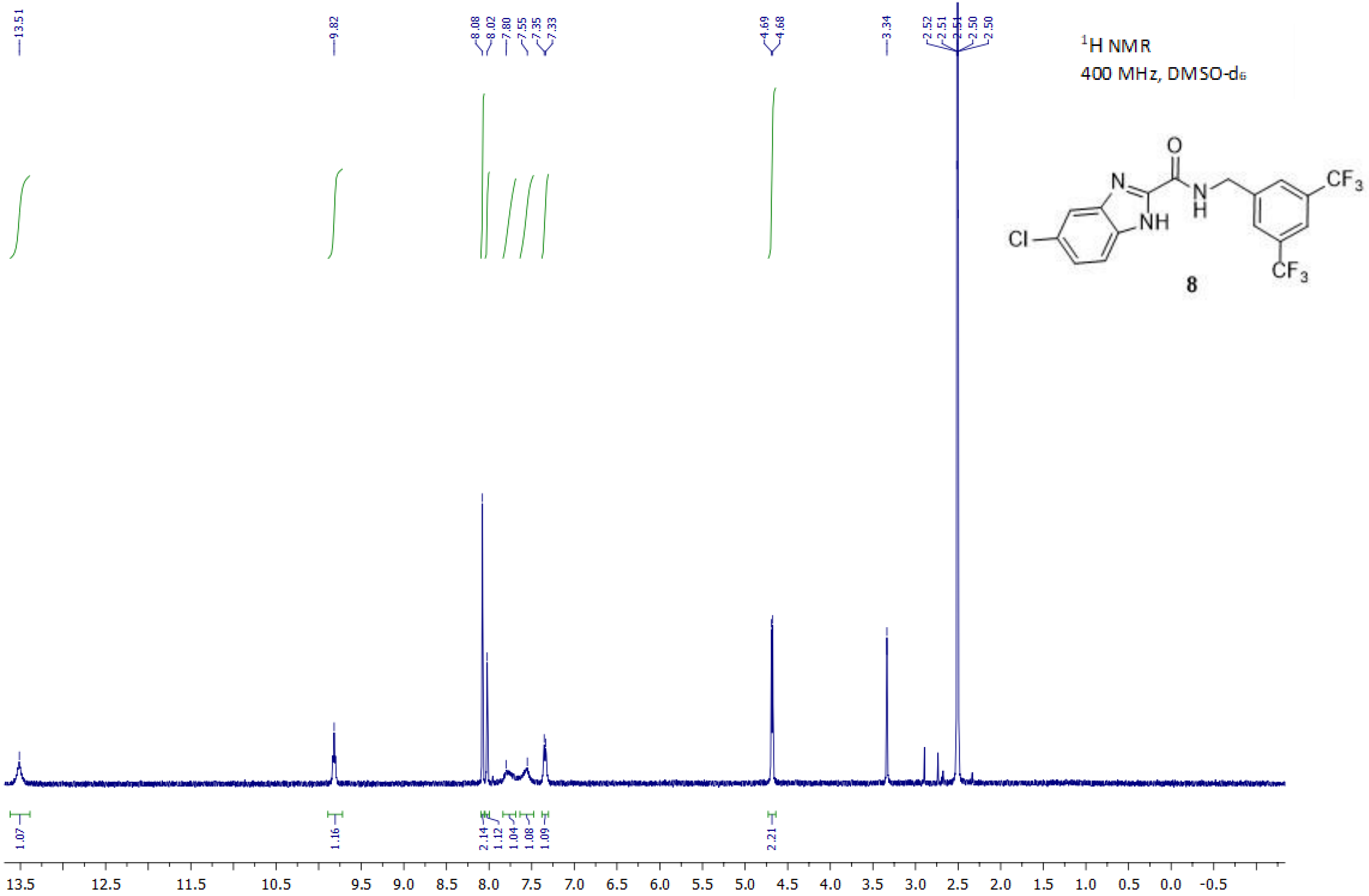

13.51
9.82
8.08
8.02
7.80
7.55
7.35
7.33
4.69
4.68
3.34
2.52
2.51
2.51
2.50
2.50
^{1}H NMR
400 MHz, DMSO-d6
O
N
NH
N
H
Cl
CF3
CF3
8
1.07
1.16
2.14
1.12
1.04
1.08
1.09
2.21
13.5
12.5
11.5
10.5
9.5
9.0
8.5
8.0
7.5
7.0
6.5
6.0
5.5
5.0
4.5
4.0
3.5
3.0
2.5
2.0
1.5
1.0
0.5
0.0
-0.5

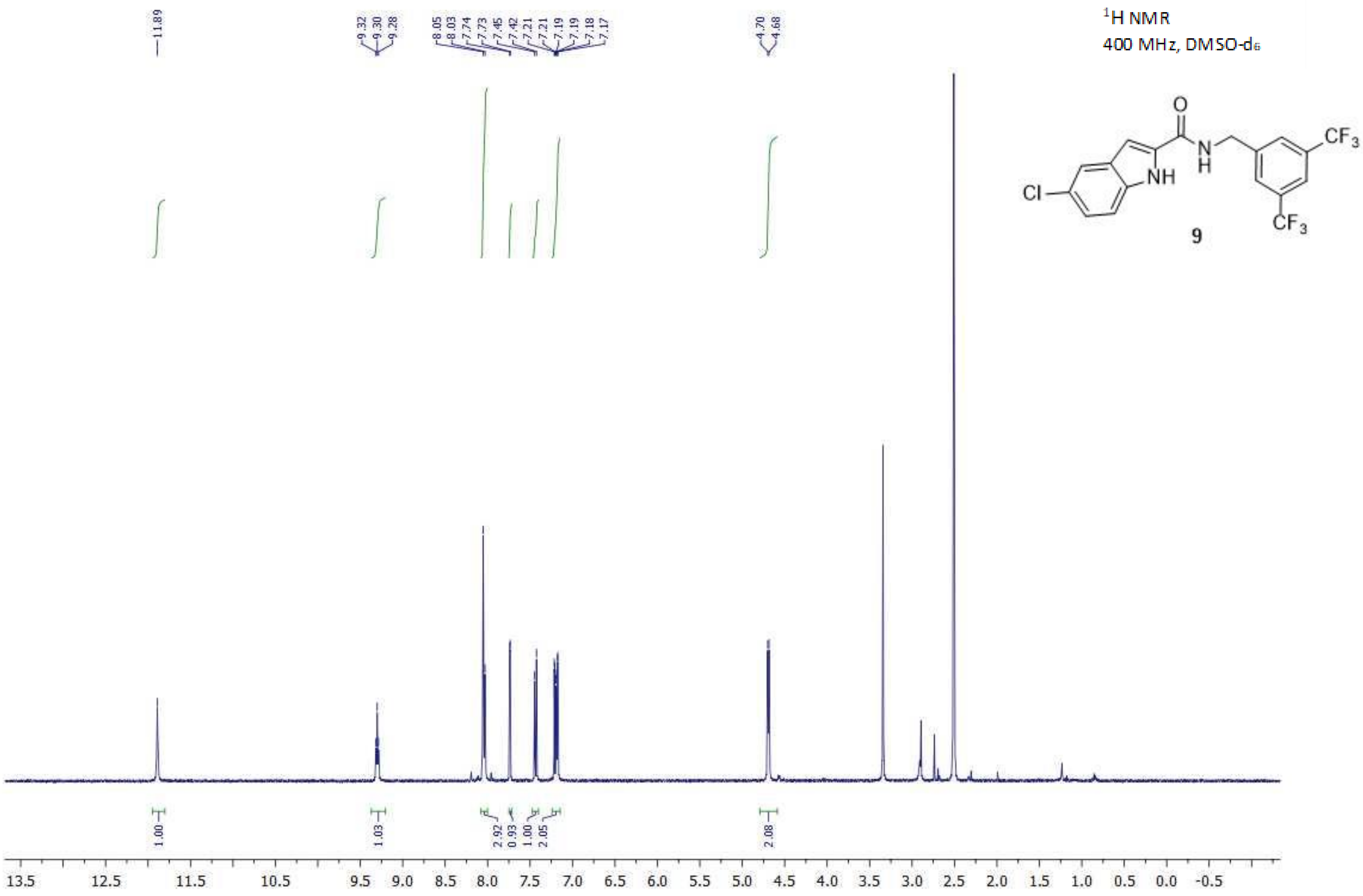

^{1}H NMR
400 MHz, DMSO-d6
9

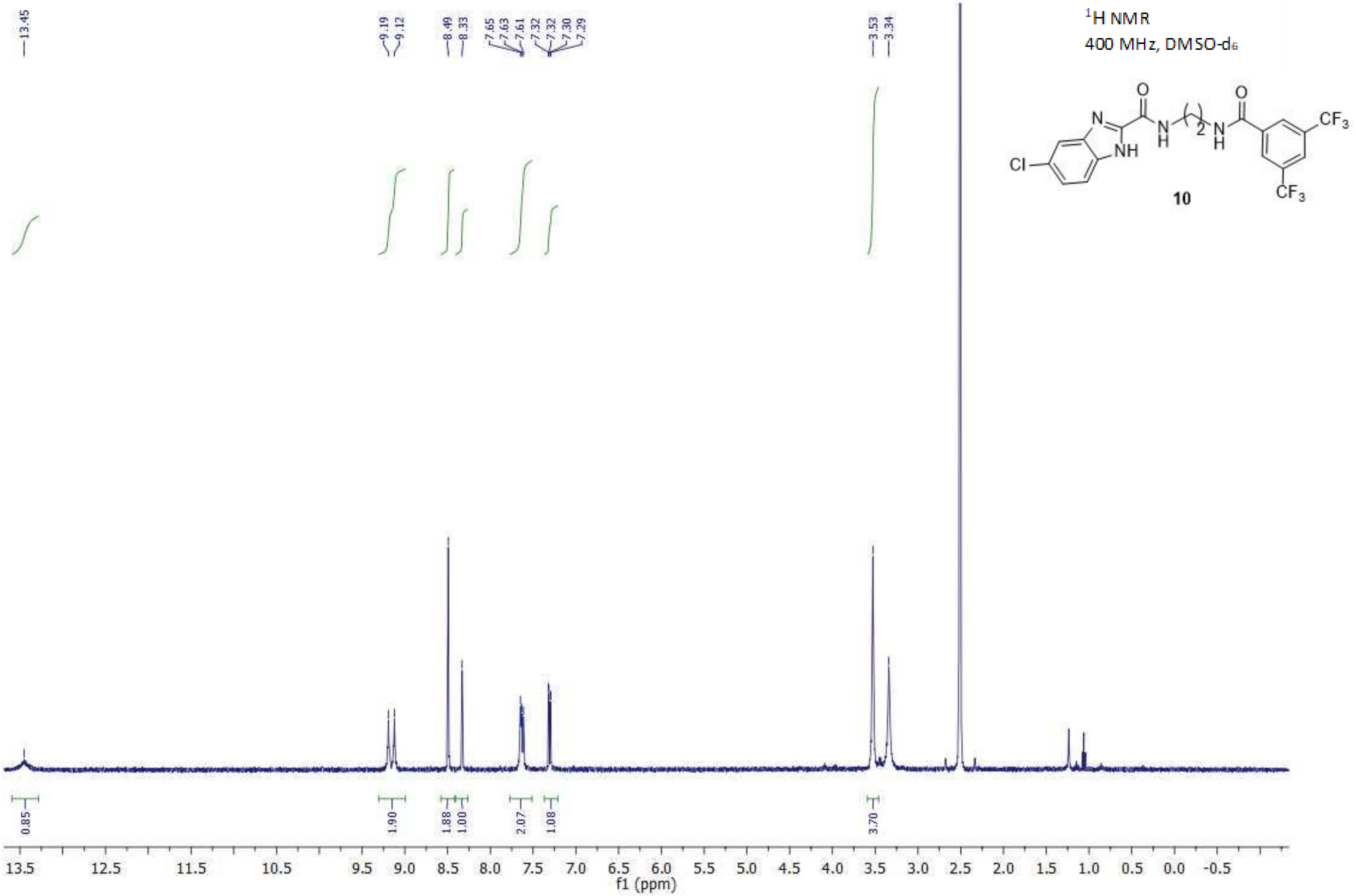
13.45
9.19
9.12
8.49
8.33
7.65
7.63
7.61
7.32
7.32
7.30
7.29
3.53
3.34
^{1}H NMR
400 MHz, DMSO-d6
Cl
N
NH
O
N
H
2
N
H
O
CF3
CF3
10
0.85
1.90
1.88
1.00
2.07
1.08
3.70
13.5
12.5
11.5
10.5
9.5
9.0
8.5
8.0
7.5
7.0
6.5
6.0
5.5
5.0
4.5
4.0
3.5
3.0
2.5
2.0
1.5
1.0
0.5
0.0
-0.5
f1 (ppm)

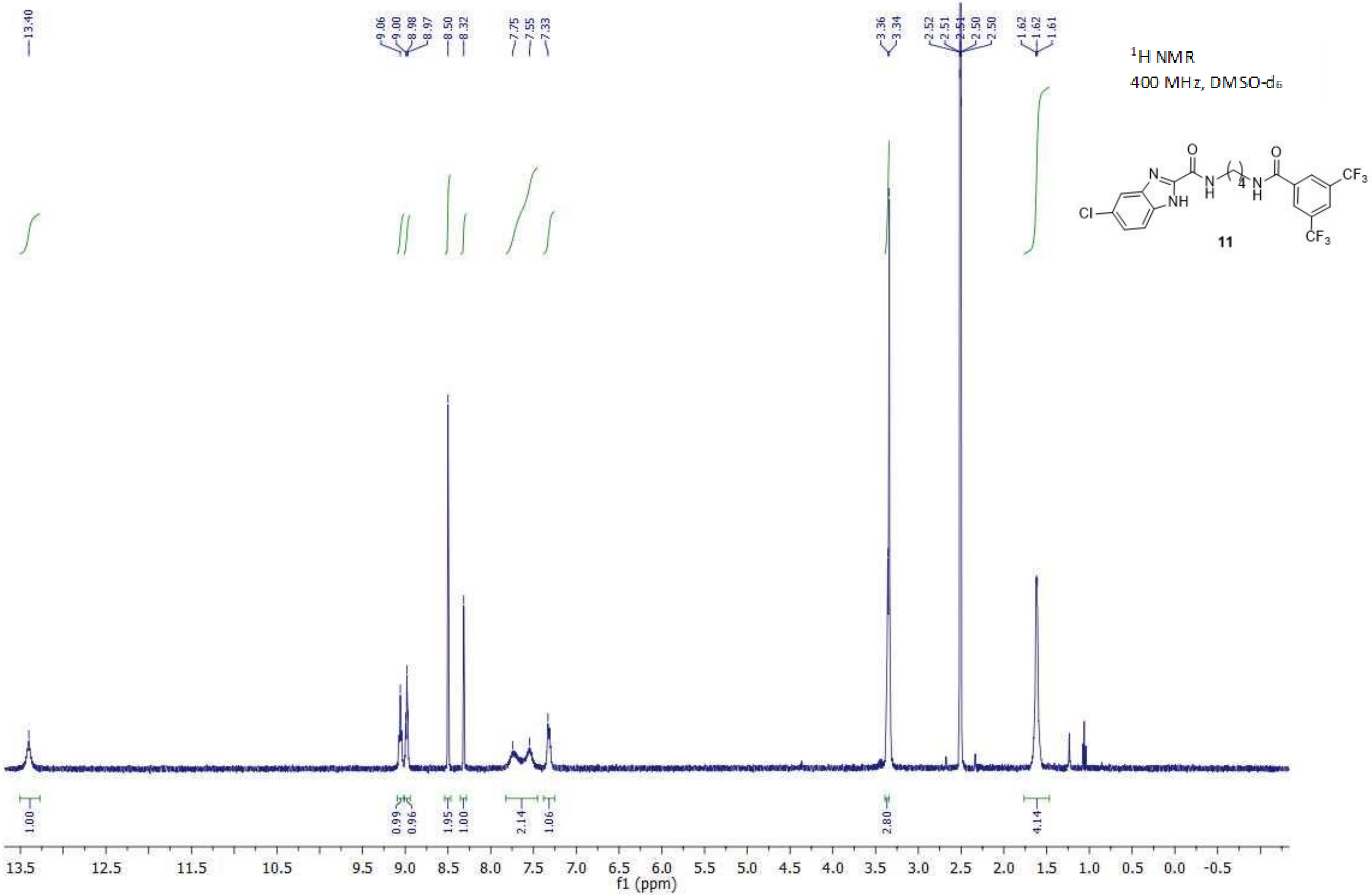

13.40
9.06
9.00
8.98
8.97
8.50
8.32
7.75
7.55
7.33
3.36
3.34
2.52
2.51
2.51
2.50
2.50
1.62
1.62
1.61
^{1}H NMR
400 MHz, DMSO-d6
Cl
N
NH
O
N
H
4
N
H
O
CF3
CF3
11
1.00
0.99
0.96
1.95
1.00
2.14
1.06
2.80
4.14
13.5 12.5 11.5 10.5 9.5 9.0 8.5 8.0 7.5 7.0 6.5 6.0 5.5 5.0 4.5 4.0 3.5 3.0 2.5 2.0 1.5 1.0 0.5 0.0 -0.5
f1 (ppm)

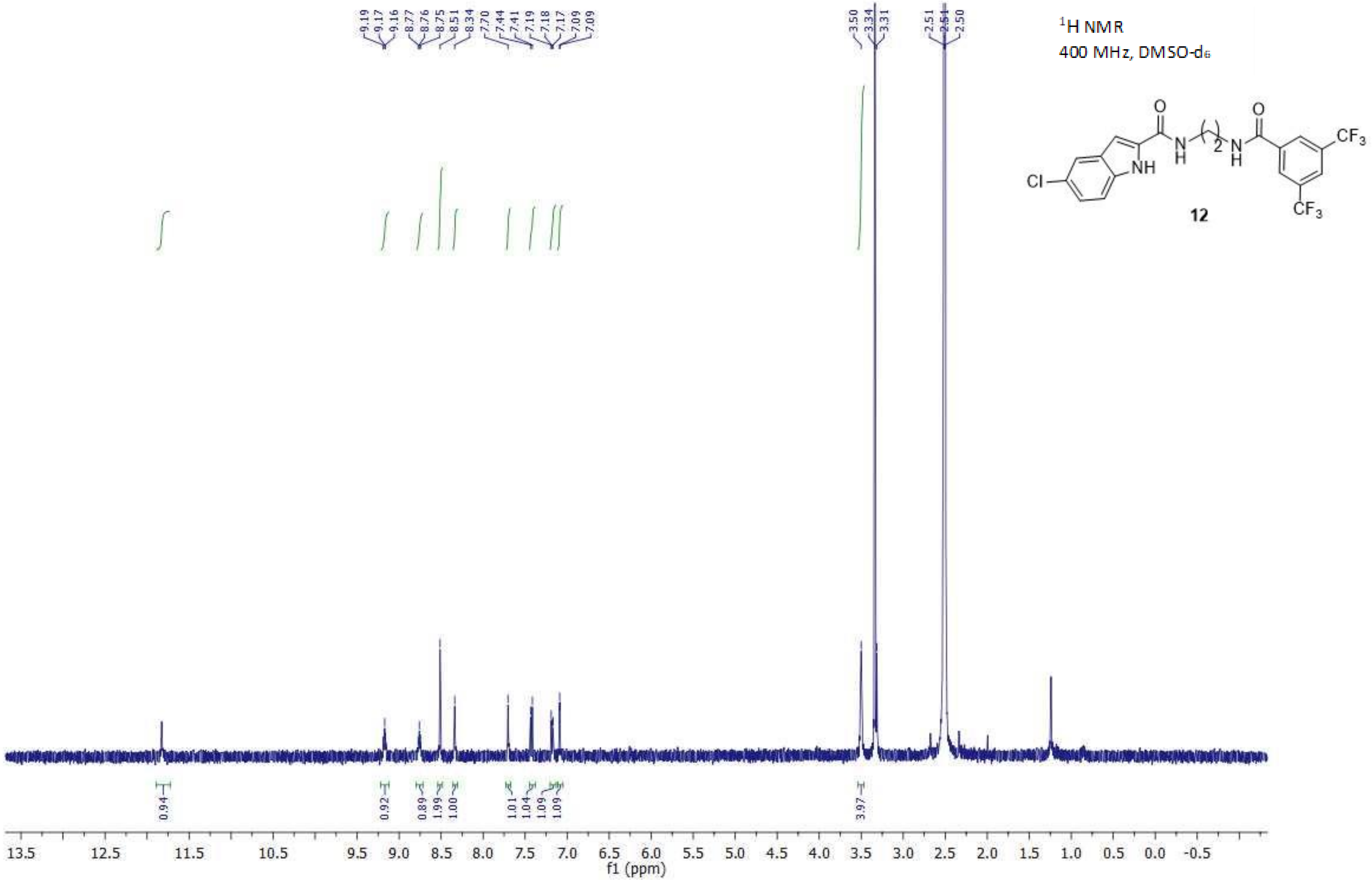

^{1}H NMR
400 MHz, DMSO-d6
12
f1 (ppm)

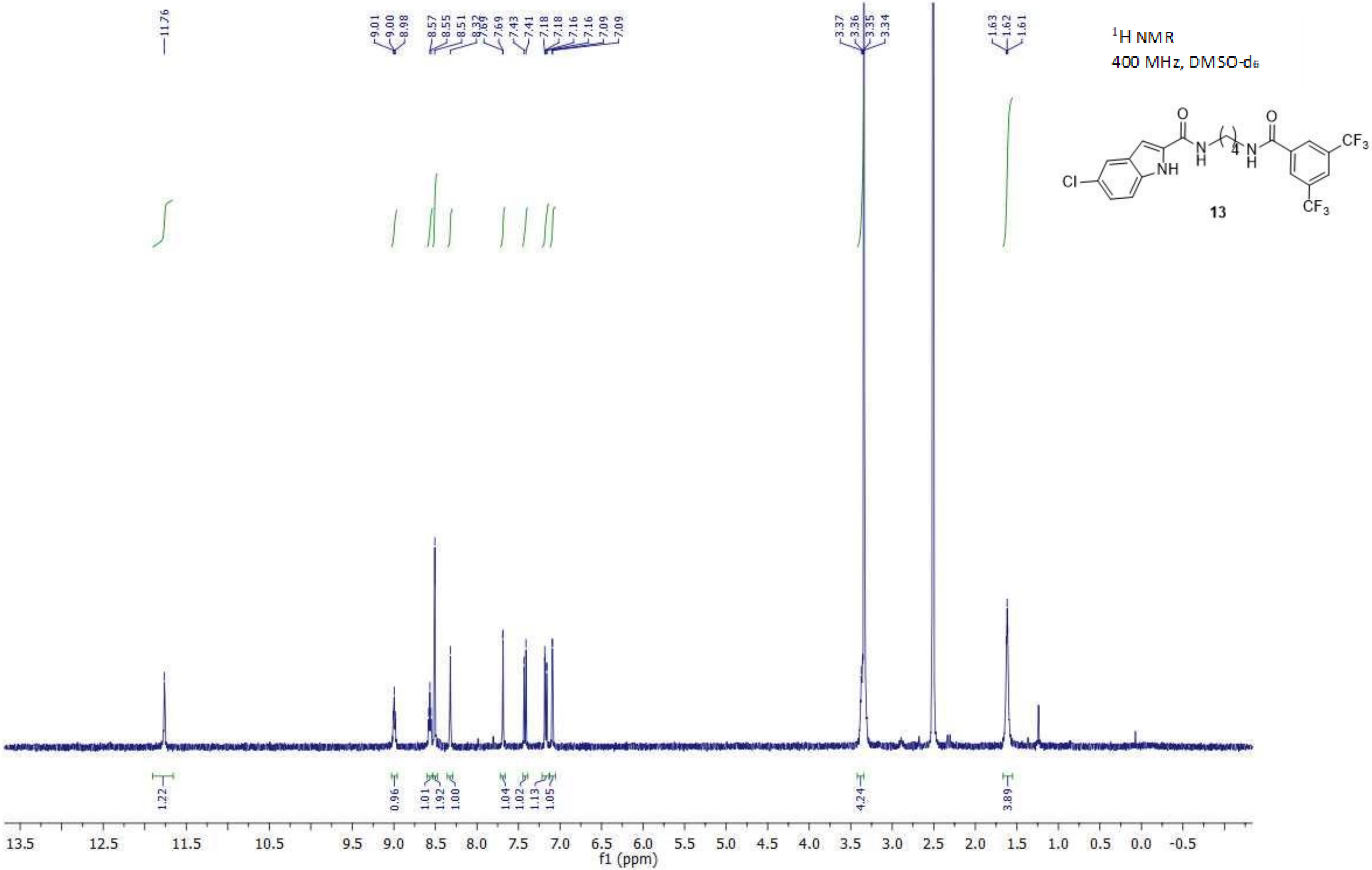

¹H NMR
400 MHz, DMSO-d6
13
f1 (ppm)
